# Techno-economic optimization, sensitivity analysis and stability evaluation of a high-renewable hybrid microgrid for rural Bangladesh

**DOI:** 10.1038/s41598-026-38328-7

**Published:** 2026-02-07

**Authors:** Diganto Biswas, Md. Feroz Ali, Mimosa Saha, Md. Shafiul Alam, Mohammad Ali, Mohammed A. AlAqil, Obaidullah Obaidi, Md. Kamrul Islam

**Affiliations:** 1 Department of Electrical and Electronic Engineering, Pabna University of Science and Technology, Pabna, 6600 Bangladesh; 2 Department of Statistics, Pabna University of Science and Technology, Pabna, 6600 Bangladesh; 3 Department of Electrical Engineering, College of Engineering, King Faisal University, Al Ahsa, 31982 Saudi Arabia; 4 Department of Energy Engineering, Faculty of Engineering, Kabul University, Kabul, 1006 Afghanistan; 5 Department of Civil and Environmental Engineering, College of Engineering, King Faisal University, Al Ahsa, 31982 Saudi Arabia

**Keywords:** Hybrid microgrid, Techno-economic optimization, Voltage–frequency stability, Sensitivity analysis, Rural electrification, Energy science and technology, Engineering

## Abstract

This study develops and evaluates a high-renewable hybrid microgrid for rural Bangladesh. The objective is to design a reliable, affordable, and grid-compliant system that supports residential, institutional, and irrigation loads. The work integrates techno-economic optimization, sensitivity analysis, and voltage–frequency stability assessment within a single framework. HOMER Pro is used to analyze multiple hybrid configurations, while MATLAB evaluates dynamic stability. The proposed contribution lies in modeling realistic field-based load profiles, incorporating converter constraints, and assessing stability across different operating conditions. A PV–wind–biogas–battery microgrid emerges as the optimal option. It achieves 88.2% renewable penetration with a net present cost of USD 206,841 and a levelized cost of energy of USD 0.0207/kWh. Solar PV and wind provide most of the annual energy, while grid support remains limited. Sensitivity analysis shows that solar and converter costs strongly influence project economics. Dynamic simulations confirm secure voltage–frequency performance and compliance with Bangladesh Grid Code limits. The results demonstrate that the proposed system offers a practical pathway for low-cost, reliable, and sustainable electrification in rural communities. The framework can also be adapted to other locations with similar resource and load characteristics.

## Introduction

In emerging economies, the link between regional development and per capita energy use has made energy-sector advancement a key national priority^1,2^. Reliable and affordable energy is fundamental to economic progress and plays a crucial role in enhancing overall productivity levels^3,4^. Over the past decade, Bangladesh has experienced a sharp increase in electricity demand, driven largely by rapid population growth and sustained economic expansion^5,6^. Meeting this rising demand remains a significant challenge for a nation of approximately 168.25 million people with one of the highest population densities globally^7,8^.

As the world’s eighth most populous country, with 1301 people per km², Bangladesh faces considerable pressure on its energy infrastructure^9,10^. Despite achieving full national electrification, the power system continues to suffer from voltage instability, frequent load shedding, and supply interruptions^11,12^. These issues undermine industrial output, reduce export competitiveness, and constrain socio-economic development^13,14^. Addressing these energy-system weaknesses is therefore vital for fulfilling the country’s long-term growth objectives^15,16^.

Bangladesh’s electricity generation capacity has grown considerably in recent years, reaching 30,277 MW, and the government plans to expand this to 31,000 MW by 2030 and 60,000 MW by 2041 as outlined in the revised PSMP 2016^17,18^. Even with this expansion, renewable energy remains essential, especially for remote rural communities where extending the national grid is costly and technically challenging^19,20^. Many donor-supported off-grid solar PV systems installed in these regions struggle to deliver long-term service because maintenance arrangements and financial provisions are often insufficient^21,22^. The country is also navigating a broader energy-resource crisis, intensified by rapid urbanization and escalating energy consumption^23,24^. Its low-lying deltaic geography and dense population present additional obstacles to developing environmentally sustainable energy systems in the context of global climate change^25,26^. Achieving national decarbonization targets will therefore require rapid expansion of renewable energy deployment. Current strategies envision renewables contributing 60% of total electricity generation by 2030 and up to 90% by 2050^27,28^. Although Bangladesh has strong potential for solar and wind development, renewables account for only about 5% of the installed generation capacity^29,30^ showing that the country is still at an early stage of its clean energy transition. This limited share indicates a heavy dependence on fossil fuels despite the clear promise of renewable technologies^31,32^. Nevertheless, recent policy attention and increased investment reflect growing national commitment to integrating more renewable energy into the grid^33,34^. Strengthening this sector is crucial for improving energy security, enhancing climate resilience, and reducing greenhouse gas emissions, all of which are central to sustainable development goals^35,36^.

Electricity demand in Bangladesh has risen sharply over the past decade. Total generation has more than doubled, and national access to electricity has increased from 47% in 2009 to 94% in 2019, largely driven by growing household consumption^37–39^. Globally, around 16% of the population still lacks reliable electricity, a barrier that limits socio-economic development and quality of life^40,41^. In Bangladesh—where nearly 65% of the population lives in rural areas—many communities continue to experience unreliable electricity supply, which slows economic progress and increases vulnerability to climate impacts^42,43^. At the same time, rising fuel prices and uncertainty in fuel imports highlight the urgency of moving away from fossil-fuel dependence^44,45^. This global and national context has encouraged a shift toward renewable energy systems as a more secure and sustainable alternative^46,47^. By December 2021, global renewable energy generation capacity had reached 3146 GW, as reported by REN21^48,49^. In Bangladesh, renewable energy development remains in a formative stage^50,51^. By 2025, the country’s total installed RE capacity stood at approximately 1,562.76 MW, with solar energy playing the dominant role at 1,268.77 MW—comprising 377.15 MW off-grid and 891.62 MW on-grid—demonstrating its broad applicability nationwide. Wind energy contributes 62.9 MW, while hydropower accounts for 230 MW, all integrated into the grid. Biogas- and biomass-based electricity production remain limited, generating only 0.69 MW and 0.4 MW, respectively^52,53^. Although solar energy has received the most policy attention, the relatively slow development of other renewable sources suggests a large untapped resource base^54,55^. Broadening and diversifying the renewable energy mix will be vital for building a more resilient and sustainable electricity system in Bangladesh^56,57^.

Rural electrification has expanded rapidly, with 76% of Bangladesh’s population residing in rural areas. Thanks to the Rural Electrification Program, the number of electrified villages increased dramatically from 250 in 1971 to 39,684, significantly improving living standards and reducing poverty rates^58^. Despite progress, long power outages—sometimes lasting more than eight hours—remain common during the hottest months of June, July, and August, revealing the system’s vulnerability to climate-related stress^59,60^. As energy demand grows, the conventional fuel-based system is becoming increasingly inadequate^61,62^. Renewable resources such as solar and wind fluctuate throughout the year due to changes in irradiance and wind speed. A hybrid renewable energy system (HRES) addresses this challenge by combining multiple renewable sources to balance these variations and provide more stable electricity generation^63,64^. Incorporating energy storage systems is also vital for balancing intermittent generation and reducing dependence on the national grid. Solar and wind resources are abundant across Bangladesh and have negligible environmental impact, making them highly suitable for decentralized power systems^65,66^. Hybrid RE-based microgrids therefore represent an optimal choice for rural electrification, particularly in areas where grid extension is economically or technically infeasible^67,68^.

A wide body of literature has examined hybrid renewable energy systems (HRES) that combine photovoltaic (PV), wind, biomass, and battery technologies to deliver reliable and sustainable electricity across varying climatic and geographical contexts. These studies differ in component configurations, optimization algorithms, and evaluation criteria, thereby offering a broad comparative foundation for assessing the techno-economic feasibility of hybrid systems. Alshammari et al. at (2018)^69^ evaluated several standalone hybrid configurations for remote pastoral communities in Saudi Arabia with an 18.67 kW peak demand. Their analysis identified the PV–biomass configuration as the most cost-effective, with a total net present cost (TNPC) of $138,521.40 and a levelized cost of energy (LCOE) of $0.099/kWh. Building upon this, Alshammari et al. at (2020)^70^ applied harmony search and particle swarm optimization techniques to hybrid standalone systems for island electrification. Their recommended wind–biomass–PV–battery design produced a higher COE of $0.254/kWh, highlighting the inherent cost challenges associated with standalone systems that operate without grid support. A more complex configuration was examined by Molu et al. at (2023)^71^ in an off-grid system for Manoka Island in Cameroon, integrating solar, wind, biogas, and hydrogen storage. With 334 residential consumers and a daily demand of 1082.9 kW, the optimized system achieved a COE of $0.1981/kWh, an internal rate of return (IRR) of 9.09%, and a payback period of 8.76 years, though the study did not explore grid-connected alternatives. Kushwaha et al. (2024)^72^ investigated socio-techno-economic-environmental (STEE) based optimal sizing of hybrid renewable energy systems for rural electrification using metaheuristic optimization techniques. Their results showed that a PV–WT–BAT–BG–DG configuration optimized using the Marine Predators Algorithm achieved the best performance, with a reported cost of energy of $0.1799/kWh. Compared to their STEE-focused sizing approach, the present study emphasizes renewable-rich microgrid optimization combined with sensitivity analysis and dynamic voltage–frequency stability evaluation, offering additional insights into operational feasibility for rural Bangladesh.

In Gaza, Al-Najjar et al. at (2022)^73^ assessed a grid-tied PV–biogas–battery hybrid system, achieving a renewable fraction of 64.3% and a COE of $0.438/kWh. Although their work did not incorporate environmental or socioeconomic analyses, it demonstrated the viability of hybrid systems under heavily constrained grid conditions. More advanced multi-source integration was explored by Sadeghi et al. (2024)^74^ in Semnan, Iran, where a PV–wind–biomass–battery system delivered a COE of $0.201/kWh and achieved a 97% reduction in CO₂ emissions. Similarly, Irshad et al. at (2024)^75^ enhanced biomass utilization through pyrolysis technology, reducing the COE to an exceptionally low $0.027/kWh while achieving a renewable energy share of 92%, underscoring the economic benefits of advanced biomass conversion pathways.

Hybrid systems have also been applied to institutional and urban contexts. In Egypt, Abdelsattar et al. at (2024)^76^ modeled a grid-connected hybrid system for Hurghada, achieving 85% renewable penetration with a competitive LCOE of $0.07/kWh, though high initial investments remained a barrier. At Marmara University in Istanbul, Aykut et al. (2020)^77^ investigated multiple PV–wind–biomass combinations using HOMER software. Their optimal configuration—1500 kW of wind and 1000 kW of biomass—resulted in an NPC of $5.62 million and a COE of $0.067/kWh, demonstrating strong potential for campus-scale renewable integration. Another university-centered study by Serat et al. (2024)^78^ assessed a PV–wind–genset–grid configuration, achieving one of the lowest reported LCOE values at $0.0172/kWh and a 94.8% renewable energy fraction. This highlights the capability of hybrid microgrids to deliver highly economical power in institutional settings. Complementary findings were reported by Kasaeian et al.^79^, who designed a grid-connected PV–diesel–biogas hybrid system and examined its performance under varying economic conditions. Their results confirmed the system’s ability to reduce emissions and dependence on diesel fuel, though biogas feedstock availability posed operational constraints. Overall, these studies collectively emphasize the critical role of hybrid systems in enhancing energy security, lowering costs, and supporting long-term sustainable development across diverse environments.

Even with the prevailing research on hybrid microgrids for rural area electrification, some significant gaps exist within the existing research work. This is because most existing research works assume a constant load with disregard for the actual variability of load in rural areas, involving both residential, educational, and irrigation purposes with a high level of seasonality. Moreover, existing research works fail to take into consideration the constraints involving the functionality of converters, which is often a determinant factor in whether the microgrid can function in reality. Moreover, dynamic stability is always evaluated for a single timescale within existing research works without considering the level of voltage-frequency support within a daily, weekly, and yearly timescale, including compliance with national grid standards. Additionally, the sensitivity level evaluated within existing research works is often incomplete, raising ambiguity about the components and outside factors within a microgrid influencing cost, reliability, and use of renewable energy sources.

As an answer to these research gaps, the aims and objectives of this study have been clearly defined. Firstly, this study aims to develop a robust modeling framework incorporating diversified levels of loading, resource characteristics, and components’ interactions relevant to the conditions existing in rural Bangladesh. Secondly, this study aims to explore the best hybrid form of a microgrid solution after conducting a thorough techno-economic analysis so that only those solutions can be considered for selection which will be technically viable and compatible with one another. Thirdly, this study aims to evaluate the dynamic performance of the optimum solution conceived within reference to variability existing within renewable sources and load conditions, considering the impact of significant techno-economic and environmental parameters.

Despite extensive research on hybrid renewable microgrids, several critical challenges remain insufficiently addressed, particularly for rural electrification in developing regions. The novelty of this study lies in its integrated and practical approach to hybrid microgrid design and validation. First, unlike most existing works that assume simplified or constant load profiles, this study develops realistic multi-sector rural load models incorporating residential, institutional, and irrigation demands based on field data, capturing strong daily and seasonal variability. Second, the proposed techno-economic optimization explicitly accounts for converter functionality and operational constraints, ensuring realistic and implementable system configurations rather than purely cost-optimal designs. Third, this work advances beyond conventional steady-state or single-timescale analyses by conducting multi-timescale voltage–frequency dynamic stability assessments under varying renewable generation and load conditions, thereby demonstrating grid-code-compliant performance. Fourth, a comprehensive sensitivity analysis is performed to identify dominant economic and technical drivers affecting system feasibility. Finally, the framework is validated through a real rural case study in Bangladesh, highlighting its applicability for cost-effective, reliable, and high-renewable rural electrification. These contributions clearly differentiate the proposed methodology from existing studies and demonstrate its advancement of current hybrid microgrid research. Although this study focuses on Nalia village, the developed modeling and optimization framework is scalable and replicable for other rural areas of Bangladesh or similar developing regions. By adjusting local load profiles, renewable resource availability, and grid conditions, the same approach can identify technically viable and economically feasible hybrid microgrid configurations. This highlights the broader applicability of the methodology beyond the specific study site.

## Materials and methods

In this study, a comprehensive analytical framework is used to evaluate different renewable scenarios. The process includes selecting the study area, analyzing load profiles, collecting resource data, designing the microgrid, performing techno-economic optimization, assessing environmental impacts, and forming the final conclusions.

### Site location

**Fig. 1:**
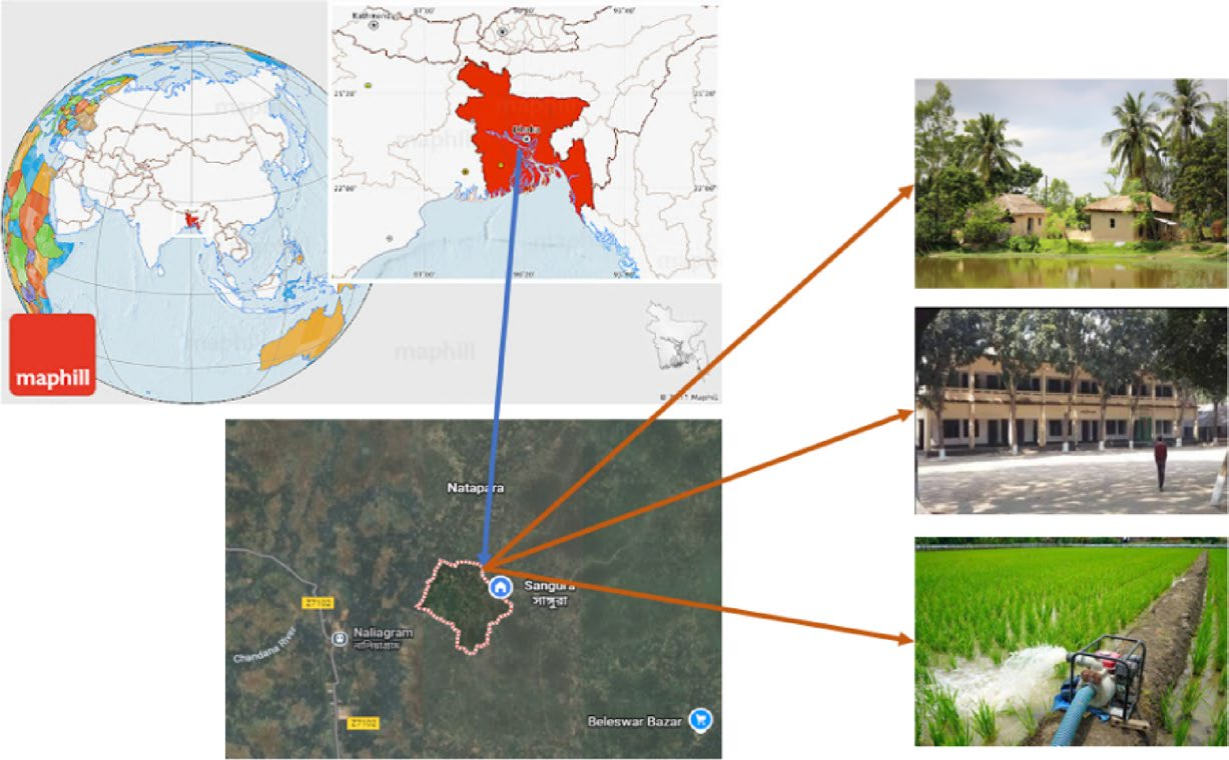
Geographical description of the study area (created by using Draw.io software, version v26.1.1).

The study was conducted in Nalia village, located under Baliakandi Thana in the Rajbari district of Bangladesh (23°36′34.5″ N, 89°37′41.0″ E). The area comprises residential settlements, educational institutions, and active agricultural land, including irrigation systems. Frequent and unplanned grid power outages adversely affect daily life, disrupt educational activities, and impede the operation of irrigation pumps. These interruptions reduce the efficiency of computer-based learning in schools and hinder agricultural productivity. To address these challenges, the research explores alternative, modern, and sustainable power sources suitable for rural applications. The objective is to enhance the stability and reliability of electricity supply for households, educational institutions, and irrigation systems. Figure 1 illustrates the geographic location of the study area created by Draw.io software—within Bangladesh, Rajbari district, and specifically Nalia village^80^. By mitigating the current energy issues, the study aims to improve educational access, support sustainable development goals, and promote socio-economic advancement for the village and its educational and agricultural infrastructure.

### Demand profile

The load data adopted in this study are structured to reflect realistic residential, community, and deferrable demand patterns, following recent socio-techno-economic-environmental based HRES studies reported in the literature^81^.Similar to these works, seasonal and time-dependent variations are incorporated to ensure practical representation of rural energy consumption^82^. Unless otherwise stated, all load profiles and figures in Sect. “Residential load profile”–“Deferrable load profile” represent aggregated community-level demand, consisting of 100 residential households, one educational institution, and irrigation load. Table 1 presents a detailed breakdown of electrical loads for three categories: Residential, Educational Institution, and Deferrable loads. For each appliance or device, the table lists the quantity, rated power, total power demand, daily operational hours, and resulting daily energy consumption. In the residential sector, common loads such as lighting, ceiling fans, refrigerators, televisions, and water pumps contribute to a total of 11.271 kWh/day per household. The educational institution category shows significantly higher energy usage—dominated by lighting, fans, PCs, and water pumps—reaching 110.22 kWh/day. The deferrable load, represented by irrigation pumping, adds 27 kWh/day. For modeling aggregated community demand, the previously calculated residential profile of 1114.5 kWh/day for 100 households aligns closely with the per-household consumption presented in this table.

**Table 1:** Load profile of residential, commercial and deferable.

	**Load description**	**Quantity**	**Power (W)**	**Total Power (W)**	**On Time (h/d)**	**Total Energy (kWh/days)**
**Residential**	Light	6	15	90	12	1.08
	Street Light	1	15	15	11	0.165
	Ceiling fan	3	60	180	8	1.44
	Refrigerator	1	300	300	24	7.2
	Television	1	80	80	8	0.64
	Water pump	1	746	746	1	0.746
					**Total =**	**11.271**
**Educational Institution**	Light	120	15	1800	8	14.4
	Ceiling fan	80	60	4800	7	33.6
	PC	40	200	8000	6	48
	Printer	5	150	750	2	1.5
	Projector	6	250	1500	4	6
	Water pump	2	1120	1120	3	6.72
					**Total =**	**110.22**
**Deferrable**	Irrigation Pumping	1	6000	6000	4.5	**27**

Explicit optimization-based load scheduling was not applied in this study due to limited demand-side automation and smart metering infrastructure in rural Bangladesh; however, load flexibility was partially captured through deferrable irrigation demand and HOMER-based dispatch optimization^83,84^.

#### Residential load profile

**Fig. 2:**
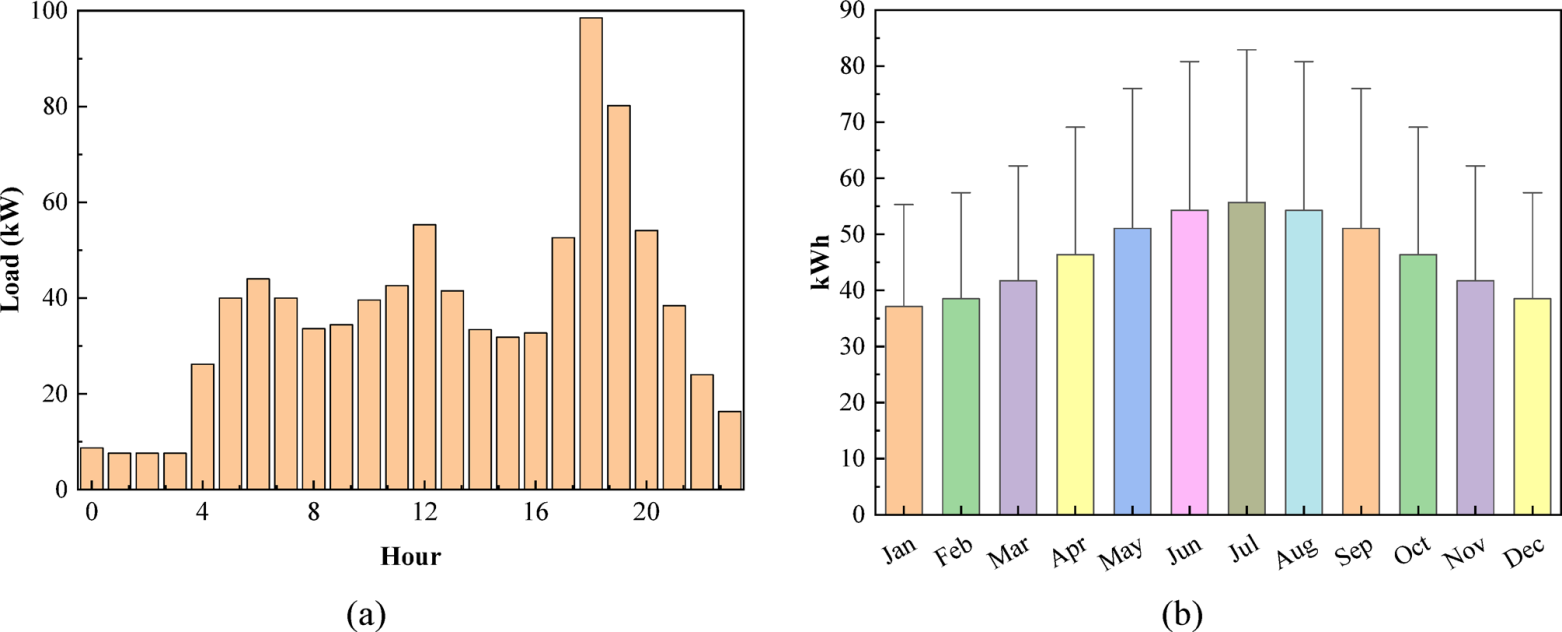
Aggregated residential load profile for 100 households: **(a)** hourly demand pattern, **(b)** monthly average energy consumption.

The monthly and hourly load characteristics provide essential insight into the energy demand patterns of the residential system. Understanding these variations helps ensure accurate system sizing, operational planning, and performance analysis for hybrid renewable energy applications. Figure 2 presents the residential load characteristics, where fig (a) illustrates the hourly load profile capturing intra-day demand variations, and fig (b) depicts the monthly average energy consumption reflecting seasonal load patterns. The monthly consumption ranges from a minimum of 1450 kWh in February to a peak of 1800 kWh in July, reflecting seasonal variations driven by temperature and cooling demand. Summer months (June–August) consistently exceed 1750 kWh, while winter months (December–February) remain between 1450 and 1550 kWh. Intermediate months such as April (1600 kWh) and September (1650 kWh) indicate moderate energy use. The hourly load curve shows low demand during early morning hours and a pronounced an evening peak approaching 100 kW for the aggregated residential load of 100 households, aligning with typical residential activity patterns. Combined, these data support accurate system sizing and energy management analysis. The monthly residential energy consumption of 1450–1800 kWh shown in Fig. 2(b) corresponds to the aggregated demand of 100 households, consistent with the daily average of 1114.5 kWh/day reported in Table 1.

#### Commercial load profile

**Fig. 3:**
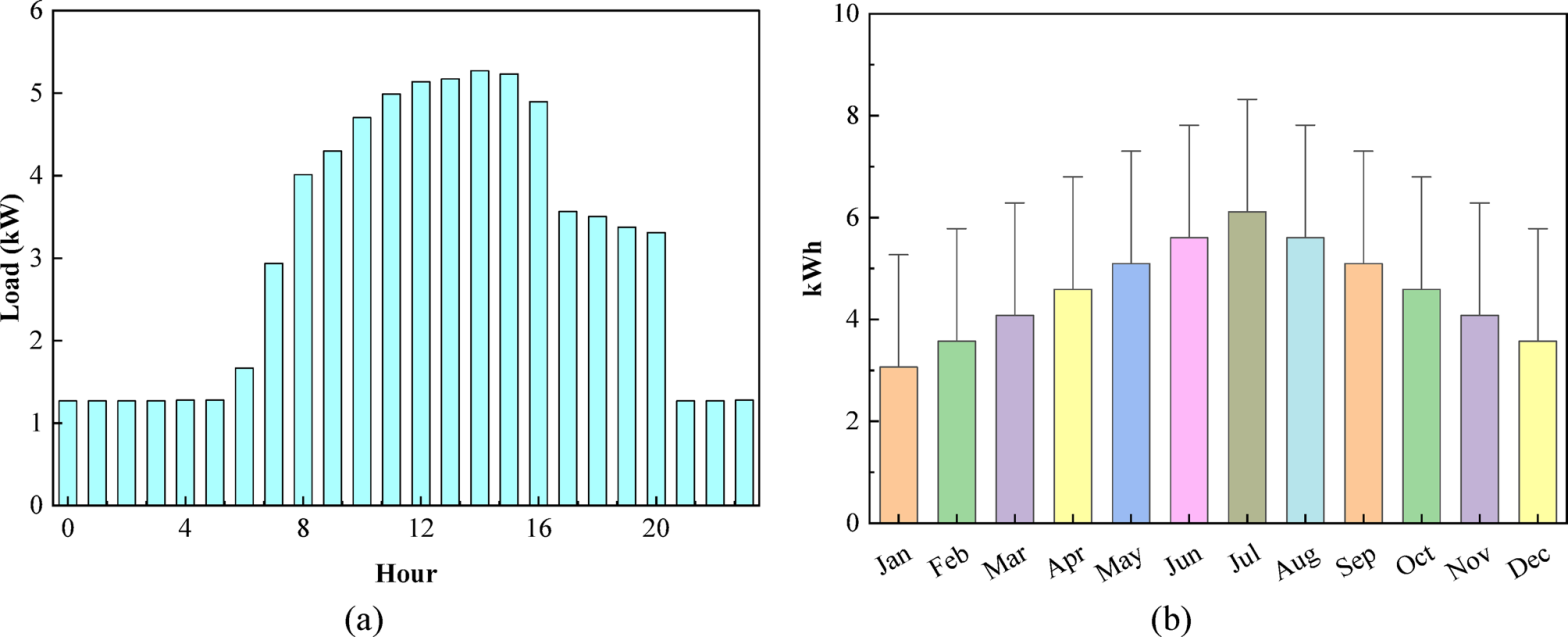
Commercial load profile: **(a)** hourly demand pattern and **(b)** Average Daily Energy Consumption (kWh/day).

Understanding the time-varying load profile is essential for accurately designing and optimizing a microgrid for educational institutions. Figure 3 presents the commercial load profile by depicting Fig. 3(a) the hourly demand pattern and Fig. 3(b) the monthly average energy consumption, effectively capturing the operational variability of the institutional facility throughout the year. The hourly load profile shows a minimum demand of 1.26 kW during late-night and early-morning hours (00:00–06:00). The load begins rising after 08:00, reaching 3.0 kW, and peaks between 12:00 and 15:00 with values ranging from 5.1 to 5.4 kW, reflecting active academic and administrative activities. The load gradually decreases after 18:00, stabilizing again around 1.3–1.5 kW at night. The monthly load plot indicates seasonal variation, with the lowest average demand in January (~ 3.0 kWh) and the highest in July (~ 6.2 kWh). Summer months (May–August) show significantly higher consumption, mainly due to intensive campus usage and cooling requirements, while winter months maintain moderate loads around 3.5–4.0 kWh.

#### Deferrable load profile

**Fig. 4:**
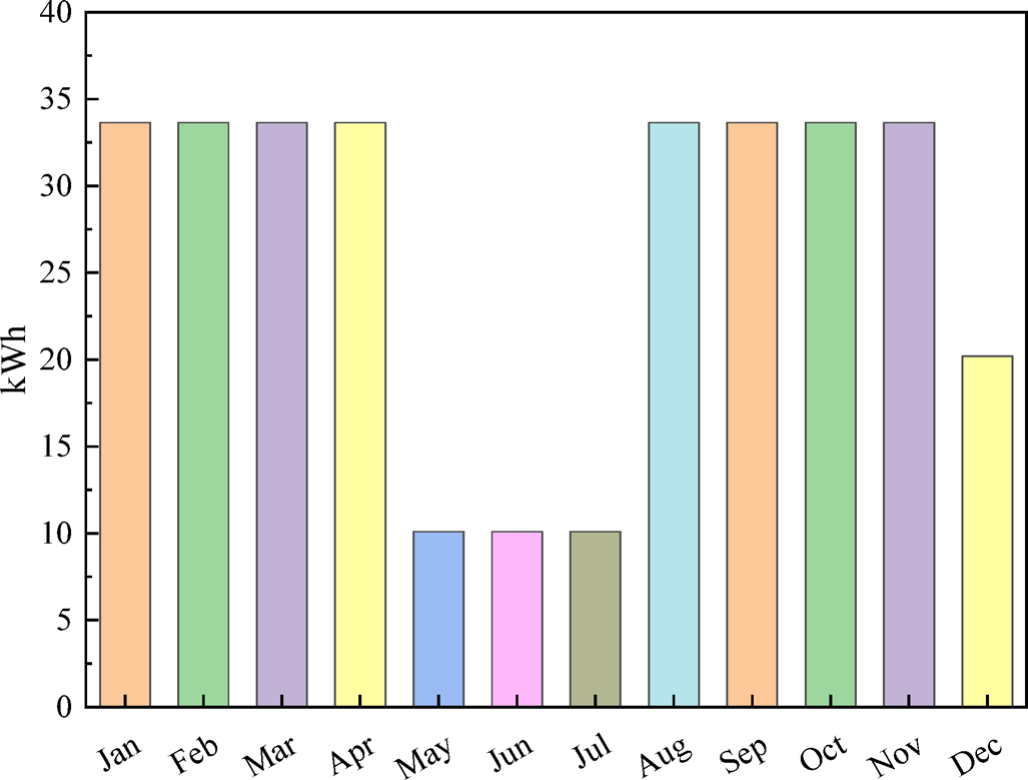
Monthly average deferrable load profile of the educational institution.

Irrigation pumps play a crucial role in Bangladesh’s agricultural cycles, where seasonal water demand strongly influences electricity consumption patterns. Understanding the monthly variation in deferrable load is essential for accurate energy planning, particularly in rural areas dependent on groundwater irrigation. Figure 4 presents the monthly average deferrable load for an irrigation pump, showing a consistent demand of 33.64 kWh from January to April, which aligns with the peak Boro rice irrigation season in Bangladesh. The load drops sharply to 10.09 kWh during May, June, and July, as the monsoon brings abundant rainfall, reducing the need for groundwater pumping. Demand rises again from August to November with the Aman cultivation period, returning to around 33.64 kWh. In December, the load decreases to 20.19 kWh because irrigation needs are lower after Aman harvesting and before the next Boro season begins.

Irrigation energy demand in rural Bangladesh varies significantly across seasons due to changes in rainfall and cropping intensity. Peak irrigation demand (33.642 kWh/day) occurs during the dry agricultural months—January to April and August to November. During the monsoon (May–July), natural rainfall reduces groundwater pumping, resulting in a substantially lower load. December shows moderate demand due to winter irrigation patterns. Using monthly seasonal factors, the annual irrigation energy consumption was computed by Eq. (1) as:

1 $$\:{E}_{\mathrm{annual\:}}=\sum\:_{i=1}^{12}\:\left({E}_{i}{D}_{i}\right)=\mathrm{9,695.62}\mathrm{k}\mathrm{W}\mathrm{h}/\mathrm{\:year}$$

The corresponding HOMER Pro scaled average deferrable load is shown in Eq. (2):

2 $$\:{E}_{\mathrm{avg\:}}=\frac{{E}_{\mathrm{annual\:}}}{365}=26.56\mathrm{k}\mathrm{W}\mathrm{h}/\mathrm{\:day}$$

To ensure one full day of irrigation autonomy, the deferrable-load storage capacity was set to 34 kWh, matching the maximum daily irrigation requirement.

### Meteorological data analysis

For the HOMER simulations, solar irradiance, clearness index, temperature, and wind speed were collected from the NASA Surface Meteorology and Solar Energy database^85^. The simulation incorporates multiple renewable resources, with corresponding datasets collected from various sources. HOMER Pro utilizes NASA’s long-term averaged climate records, typically covering a 22-year period (1983–2005) as its default dataset for temperature, wind speed, and solar radiation, which are then spatially averaged for the selected site^86,87^.

#### Solar irradiation and clearness index

**Fig. 5:**
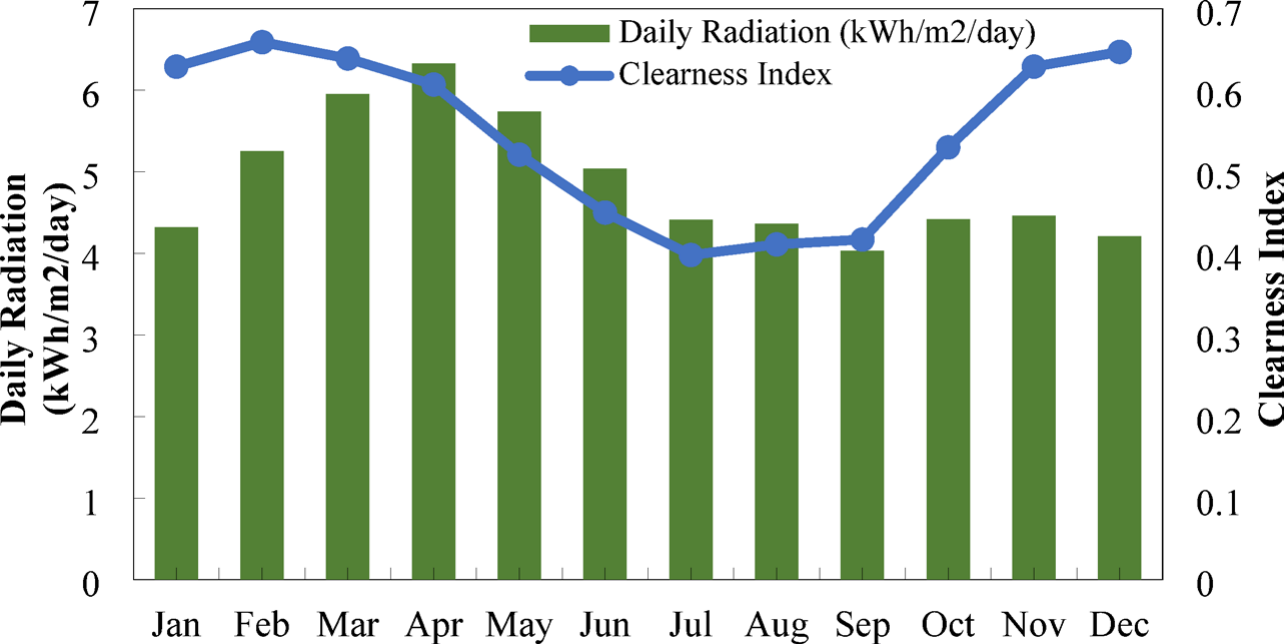
Solar irradiation and clearness index for the site.

Figure 5 depicts the monthly fluctuation of clearness index and daily solar radiation at Nalia village, Baliakandi Upazila, Rajbari District. The clearness index ranges from 0.384 in July to 0.635 in December, indicating seasonal fluctuations in atmospheric transparency. Correspondingly, daily radiation peaks at 5.90 kWh/m²/day in April and 5.60 kWh/m²/day in March, while the lowest values occur in September (4.02 kWh/m²/day) and July–August (4.24 kWh/m²/day). The combined trends demonstrate strong solar availability during spring and early summer, with moderate resource levels in winter and reduced radiation during the monsoon months. In respect to this variability, solar energy systems should consider this, therefore having supplemental sources of energy from either wind or biogas whenever the radiation is low^88^.

#### Temperature

**Fig. 6:**
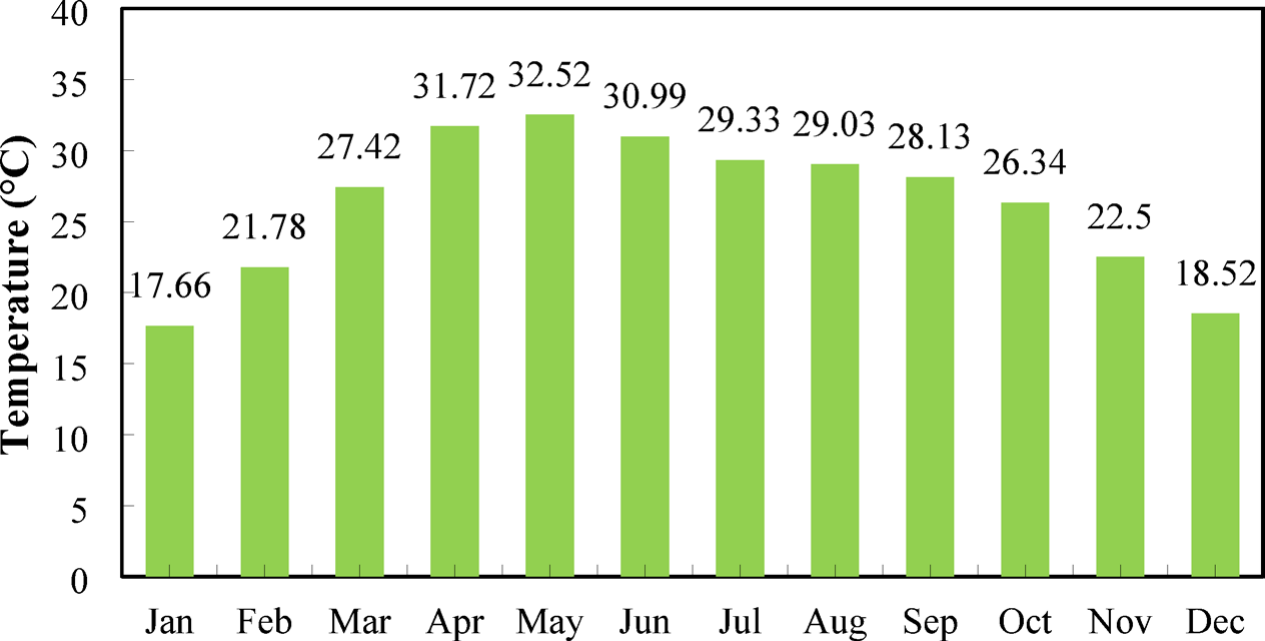
Monthly temperature variation for the site.

Figure 6 represents Nalia village, Baliakandi Upazila, Rajbari District monthly average temperature profile. Temperatures rise steadily from 18.34 °C in January to a peak of 31.27 °C in May, indicating the warmest period of the year. April also exhibits a high temperature of 31.18 °C, reflecting early summer conditions. From June onward, temperatures gradually decline, with 29.94 °C in June, 28.87 °C in July, and 28.64 °C in August, demonstrating a slow cooling trend. The transition into autumn is marked by moderate temperatures, including 27.97 °C in September and 26.56 °C in October. The climatic profile of the region follows a typical subtropical pattern with hot summers and mild winters, with the coolest months being November and December at 23.06 °C and 19.28 °C, respectively. Temperature increases affect PV efficiency by elevating the internal resistance of the cells^89^.

#### Wind speed

**Fig. 7:**
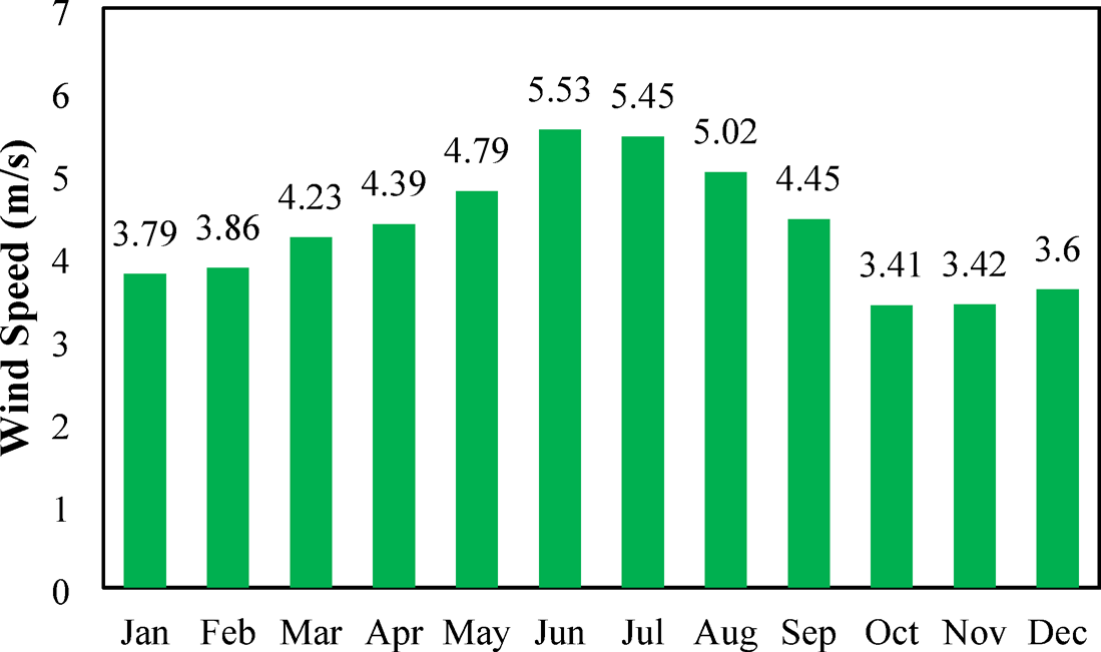
Seasonal Wind Speed Fluctuations for the site.

Figure 7 illustrates monthly average wind speed variation in Nalia village, Baliakandi Upazila, Rajbari District. Wind speeds are lowest during the cooler months, beginning with 3.75 m/s in January and remaining relatively mild through March (4.12 m/s). A steady increase follows in spring, reaching 4.86 m/s in April and 5.15 m/s in May. The highest wind speeds occur during early to mid-summer, peaking at 5.76 m/s in June and 5.86 m/s in July, indicating the period of strongest wind resource availability. After July, wind speeds gradually decline, dropping to 5.25 m/s in August and 4.45 m/s in September. The weakest wind conditions occur in late autumn, with 3.32 m/s in October and 3.21 m/s in November, followed by a slight rise to 3.41 m/s in December. Overall, the figure shows that summer months provide the most favorable wind conditions, a key consideration for wind energy potential and hybrid renewable system planning^90^. Wind turbine output similarly depends directly on wind speed^91^.

### Biomass resource

**Fig. 8:**
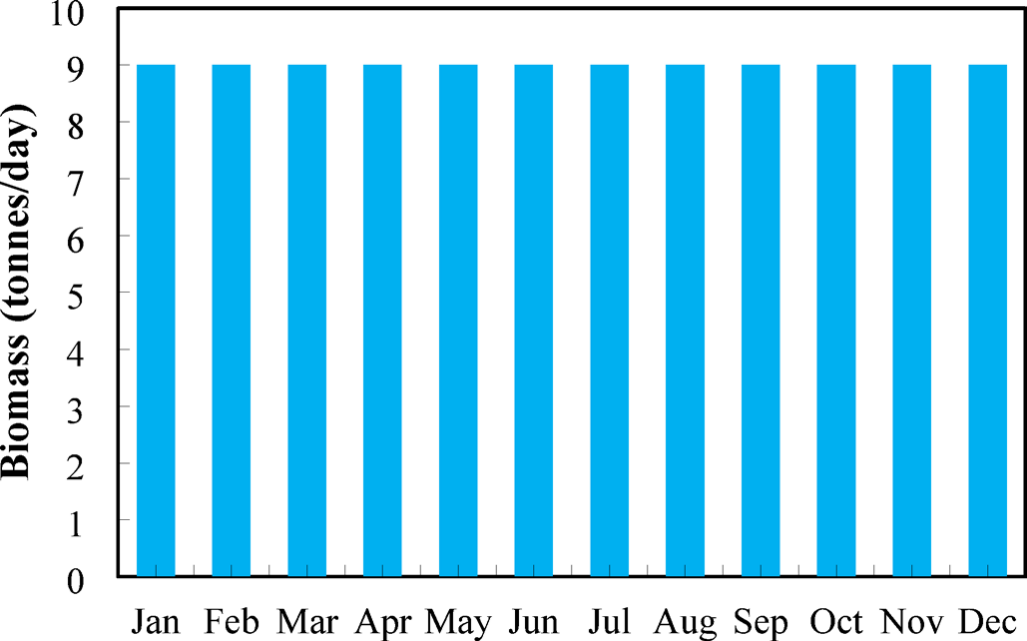
Sustainable biomass availability per month for the site.

Figure 8 shows monthly biomass availability at Nalia village, Upazila Baliakandi, Rajbari District, with a uniformly distributed supply of 9 tonnes/day for each month of the year. The selection of 9 tonnes/day of biomass is based on a realistic estimate of what a rural Bangladeshi village like Nalia can sustainably supply each day. Most of this biomass comes from everyday kitchen waste, cow dung, and small agricultural residues. On average, a rural resident generates around 1.3–1.5 kg of usable organic waste per day, including food scraps, crop leftovers, and livestock manure. For a community of roughly 6,000 people, this amounts to about 8.4 tonnes/day of recoverable biomass. In addition, small farming activities—such as rice milling, vegetable waste, and seasonal crop by-products—typically add another 0.5–0.7 tonnes/day. Together, these sources provide a stable supply close to 9 tonnes/day, which is why this value was used. It represents a dependable and sustainable estimate that avoids overestimating the resource while ensuring enough fuel for hybrid renewable power generation throughout the year.

### Modeling the components

All mathematical models used in this study are based on standard formulations implemented in HOMER Pro and widely reported in the literature. For clarity and rigor, all variables, parameters, and assumptions associated with each equation are explicitly defined, and the physical significance of each model is briefly explained.

#### Solar PV system

PV cells work by transforming sun energy into electrical power using semiconductor devices, with silicon-based material being utilized as the primary material in the cells^92^. PV power output depends on the rated capacity, derating factors, solar radiation, and temperature effects, as expressed in (3)^93^, The cell temperature, which influences PV performance, is determined using the relation in (4)^94,95^. Cell temperature significantly affects efficiency; higher temperatures generally reduce power output^96,97^.

3 $$\:{P}_{output}^{PV}={P}_{rated}^{PV}\times\:{F}_{D}^{PV}\times\:\left(\raisebox{1ex}{${G}_{T}$}\!\left/\:\!\raisebox{-1ex}{${G}_{T,STC}$}\right.\right)\times\:[1+{\eta\:}_{P}\left({T}_{c}-{T}_{c,STC}\right)]$$

4 $$\:{T}_{c}=\frac{{T}_{a}+\left({T}_{c,NOCT}-{T}_{a,NOCT}\right)\left(\frac{{G}_{T}}{{G}_{T,NOCT}}\right)[1-\frac{{\eta\:}_{mp,STC}\left(1-{\alpha\:}_{p}\times\:{T}_{c,STC}\right)}{{\tau\:}_{a}}]}{1+({T}_{c,NOCT}-{T}_{a,NOCT})\left(\frac{{G}_{T}}{{G}_{T,NOCT}}\right)\left(\frac{{\alpha\:}_{p}{\eta\:}_{mp,STC}}{{\tau\:}_{a}}\right)}$$

Here, $\:{P}_{\mathrm{output}}^{PV}$ represents the actual electrical power generated by the PV array (W), while $\:{P}_{\mathrm{rated}}^{PV}$ denotes its nominal power under standard test conditions (W). The term $\:{F}_{D}^{PV}$ indicates the overall derating factor that accounts for system losses such as dust accumulation and wiring resistance. $\:{G}_{T}$ is the solar irradiance incident on the PV surface (W/m²), and $\:{G}_{T,STC}$ refers to the STC irradiance level, typically 1000 W/m². The parameter $\:{\eta\:}_{P}$ is the temperature coefficient of power (%/°C). $\:{T}_{c}$ and $\:{T}_{c,STC}$ denote the actual and STC cell temperatures (°C), respectively. $\:{T}_{a}$ is the ambient temperature (°C). $\:{T}_{c,NOCT}$ is the module cell temperature at NOCT (around 45 °C), with $\:{T}_{a,NOCT}$ as the corresponding ambient temperature (usually 20 °C), and $\:{G}_{T,NOCT}$ the irradiance at NOCT (about 800 W/m²). Furthermore, $\:{\eta\:}_{mp,STC}$ is the module efficiency at STC, $\:{\alpha\:}_{p}$ is the temperature coefficient of power loss (1/°C), and $\:{\tau\:}_{a}$ denotes the transmittance–absorptance product.

#### WT model

Wind turbine modeling in HOMER is based on hourly wind data, scaled to hub height using either the power law or logarithmic law, given in (5) and (6)^98,99^, and the logarithmic law method, preferred for complex terrains where higher precision is required and planned in (6)^78^. Adjustments for air density and system losses are included through the equations in (7), (8), and (9)^100,101^.

5 $$\:{U}_{hub}^{W}={U}_{anem}^{W}\times\:{\left(\frac{{Z}_{hub}^{W}}{{Z}_{anem}^{W}}\right)}^{\alpha\:}$$

6 $$\:{U}_{hub}^{W}={U}_{anem}^{W}\times\:\left(\frac{\mathrm{ln}\left(\frac{{Z}_{hub}^{W}}{{Z}_{o}}\right)}{\mathrm{ln}\left(\frac{{Z}_{anem}^{W}}{{Z}_{o}}\right)}\right)$$

7 $$\:{P}_{output}^{W}={P}_{output,STC}^{W}\times\:\left(\frac{\rho\:}{{\rho\:}_{o}}\right)-{P}_{TL}^{W}$$

8 $$\:{P}_{output,STC}^{W}=\left\{\begin{array}{c}{P}_{rated}^{W}\times\:\left(\frac{{U}_{t}^{W}-{U}_{in}^{W}}{{U}_{r}^{W}-{U}_{in}^{W}}\right)\:\:\:\:\:\:if\:{U}_{in}^{W}\le\:{U}_{t}^{W}\le\:{U}_{r}^{W}\\\:{P}_{rated}^{W}\:\:\:\:\:\:\:\:\:\:\:\:\:\:\:\:\:\:\:\:\:\:\:\:\:\:\:\:\:\:\:\:\:\:\:\:if\:{U}_{r}^{W}\le\:{U}_{t}^{W}\le\:{U}_{out}^{W}\\\:0\:\:\:\:\:\:\:\:\:\:\:\:\:\:\:\:\:\:\:\:\:\:\:\:\:\:\:\:\:\:\:\:\:\:\:\:\:\:\:\:\:\:\:\:\:\:if\:{U}_{in}^{W}>{U}_{t}^{W}\:\:\:\:\:\:\:\:\:\:\:\:\:\:\:\:\end{array}\right.$$

9 $$\:{P}_{TL}^{W}={P}_{A}^{W}+\:{P}_{P}^{W}+\:{P}_{En}^{W}+\:{P}_{Wa}^{W}+\:{P}_{E}^{W}+\:{P}_{C}^{W}\:$$

#### Biogas system

Biomass in the form of agricultural residues, wood waste, and livestock or human waste is also a widely available resource in rural Bangladesh. Biogas generated from these materials contains methane and carbon dioxide^102^. In this study, a 60 kW generator is incorporated to support the system when solar and grid supply fall short. Livestock manure availability is estimated using Eqs. (14), (11), and (12)^103,104^.

10 $$\:M=\sum\limits_{n=1}^{i}\:\left({N}_{i}\times\:{m}_{i}\right)$$

where M is the amount of manure produced in one year (tons), N_i_ is the total number of animals, m_i_ is manure produced by a single animal, n is the number of specific group of animals,

11 $$\:{E}_{B}=\sum\limits_{n=1}^{i}\:\left({N}_{i}\times\:{m}_{i}\times\:{k}_{DMi}\times\:{K}_{OMi}\times\:{v}_{Bi}\times\:{e}_{Bi}\right)$$

V_b_ is the biogas volume per year (m^3^) from livestock manure, K_Dmi_ is dry contents in manure, K_Omi_ is organic contents in dry material, _Bi_ is specific biogas output (m^3^/tons),

12 $$\:P=\frac{{E}_{B}}{{K}_{e}\times\:{T}_{c}}$$

while P is the biomass energy generation (kW), K_e_ is the coefficient of plant efficiency usually 0.4, T_c_ is yearly operation hours of plant. The biomass electricity generation can be estimated by using following Eq. (13).

13 $$\:{E}_{BM}=\frac{T{F}_{a}\times\:1000\times\:C{V}_{BM}\times\:{\eta\:}_{BM}}{860\times\:\left(\frac{\mathrm{\:operating.hours\:}}{\mathrm{\:day\:}}\right)}$$

#### Converter

In hybrid AC/DC systems, converters play a critical role in maintaining efficient power flow, with losses represented through their conversion efficiency as shown in (14)^105–107^.

14 $$\:{P}_{out}^{Con}={P}_{in}^{Con}\times\:{\eta\:}_{Con}$$

#### Battery storage

The battery energy storage system is another key component, and its state of charge varies depending on charging or discharging conditions, calculated using (15)^108,109^:

15 $$\:\mathrm{S}\mathrm{O}\mathrm{C}\left(\mathrm{t}\right)\:=\:\mathrm{S}\mathrm{O}\mathrm{C}(\mathrm{t}\:-1)\:\times\:{\int\:}_{\mathrm{t}-1}^{\mathrm{t}}\frac{{{\upeta\:}}_{\mathrm{b}\mathrm{a}\mathrm{t}}\:\times\:\:{\mathrm{L}}_{\mathrm{b}}\left(\mathrm{t}\right)\:}{{\mathrm{V}}_{\mathrm{b}\mathrm{u}\mathrm{s}\:}}\mathrm{d}\mathrm{t}$$

Where, $\:{\eta\:}_{bat}$ is Battery efficiency [%], L_b_(t) is Load power of the battery [kW], V_bus_ is Bus voltage [volt].

#### Utility grid integration

According to the Bangladesh Power Supply Regulatory Commission, the grid purchase price was set at $0.08/kWh, and the sell-back price was $0.04/kWh, as used in HOMER Pro^110^. During electricity shortages, the grid supplies the required energy. HOMER calculates the cumulative yearly energy charge using the following Eq. (22)^10,111^.

16 $$\:{C}_{AEC}=\sum\limits_{x}^{\mathrm{rates\:}}\:\sum\limits_{y}^{12}\:{E}_{gp,x,y}{P}_{\mathrm{power\:},x}-\sum\limits_{x}^{\mathrm{rates\:}}\:\sum\limits_{y}^{12}\:{E}_{gs,x,y}{P}_{\mathrm{sellback\:},x}$$

HOMER utilizes the following equation to determine the total annual grid demand charge (listed after December) by Eq. (17):

17 $$\:{C}_{gd}=\sum\limits_{x}^{\mathrm{rates\:}}\:\sum\limits_{y}^{12}\:{P}_{pgd,x,y}{D}_{x}$$

A real discount rate of 15% and an inflation rate of 9% were applied in the HOMER Pro simulations. In the base case, grid export was assumed to be permitted up to the interconnection capacity without curtailment penalties; this assumption represents a modeling simplification and is discussed as a limitation.

### Economic modelling

Economic analysis in HOMER is a key element of the assessment of bankability and cost-effectiveness for varying combinations of energy generation measures^112^. By evaluating the cost and benefits from various system alternatives, economic modeling supports stakeholders in this field to take investment decisions in clean and sustainable energy technologies^113^.

#### NPC

Economic modelling is essential for understanding the long-term viability of different energy system configurations. HOMER evaluates key financial indicators, including NPC in Eq. (18)^114^:

18 $$\:NPC=\frac{{C}_{ann,total}}{CRF(i,\:{R}_{proj})}$$

where$\:,\:\:{C}_{ann,total}\:$is the total cost per annum and CRF is the capital recovery factor, i is the rate of interest in%, $\:{R}_{proj}$ is life of the project in years. Capital recovery factor is a multiplier by which present value of an annuity (a series of equal annual cash flows) can be determined. The value of CRF is determined with the aid of the following formula (19)^115^

19 $$\:CRF\left(i,\:N\right)=\frac{i{(1+i)}^{N}}{{(1+i)}^{N}-1}$$

where N is the number of years and i is calculated employing Eq. (20)^116^:

20 $$\:i=\frac{{i}_{o}-f}{1+f}$$

where, $\:{i}_{o}\:$is the nominal interest rate and f is the annual inflation rate.

#### LCOE

Economic modelling is essential for understanding the long-term viability of different energy system configurations. HOMER evaluates key financial indicators, including LCOE in Eq. (21)^117^:

21 $$\:LCOE=\frac{{C}_{ann,total}}{\:{E}_{prim,AC}+{E}_{prim,DC}+{E}_{grid,sale}}$$

Where, $\:{C}_{ann,total}$ is the annual total cost, $\:{E}_{prim,AC}$ is the AC primary load supplied, $\:{E}_{prim,DC}$ is the DC primary load supplied and $\:{E}_{grid,sale}$ is the total grid sales.

#### IRR

Economic modelling is essential for understanding the long-term viability of different energy system configurations. HOMER evaluates key financial indicators, including IRR in Eq. (22)^118^.

22 $$\:IRR={\sum\:}_{i=0}^{N}\frac{N{C}_{i}}{(1+IRR)}$$

The parameters under consideration match the values found in the NPC formula. After deducting the production costs, the return increases with the IRR^119^.

#### RF

The ratio of energy produced by renewable sources to the overall energy produced by the system is known as RF. It is determined by Eq. (23) and has no dimensions^120^:

23 $$\:RF=1-\frac{{E}_{nr}+{H}_{nr}}{{E}_{s}+{H}_{s}}$$

Where, $\:{E}_{nr}$ is the non-renewable generation of electricity (kWh/yr), $\:{H}_{nr}\:$is the non-renewable generation of heat (kWh/yr), $\:{E}_{s}\:$is the total served electrical load (kWh/yr) and $\:{H}_{s}$ is the total served thermal load (kWh/yr).

All models and parameters used in this study are consistent with standard HOMER Pro implementations and peer-reviewed literature, ensuring mathematical validity and reproducibility of results.

### HOMER pro

**Fig. 9:**
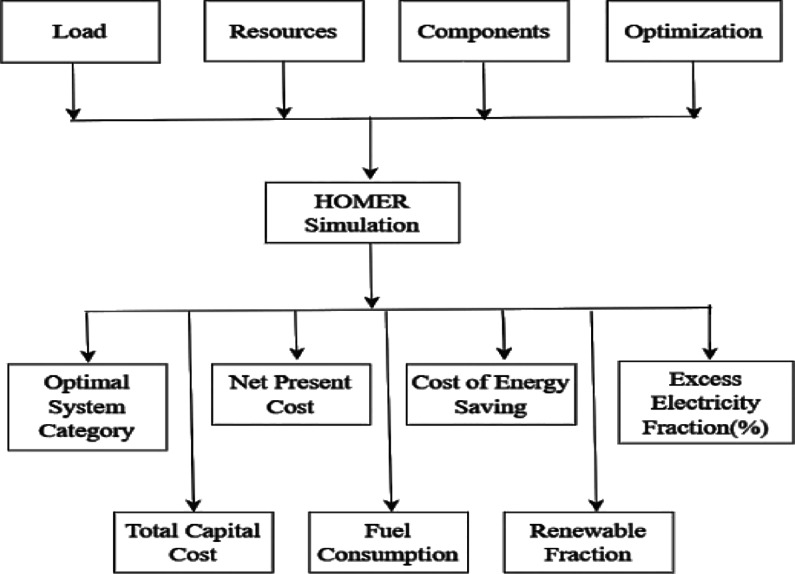
Architecture of HOMER Pro software.

HOMER Pro (version 3.14.2) is one of the most widely used simulation and optimization tools for designing hybrid renewable energy systems. Developed originally by the National Renewable Energy Laboratory (NREL), it has become a standard platform for engineers, researchers, and policymakers working with distributed power systems^121,122^. Its strength lies in its ability to model the technical feasibility and economic performance of complex microgrid architectures, helping users identify the most suitable energy mix for remote or grid-constrained areas^123^. The software supports a broad range of renewable and conventional technologies, including PV, wind turbines, battery storage, biomass generators, and even advanced options such as hydrogen systems^124^. HOMER Pro evaluates system configurations based on inputs such as local resource availability, load demand, component characteristics, and operational constraints. It then optimizes the design using key economic indicators—mainly NPC and COE—to determine the most cost-effective and reliable solution^125^. Figure 9 illustrates the general architecture of HOMER Pro, highlighting how users can explore different system combinations depending on budget limits and technical requirements^126,127^. The load, resources, components, and optimization criteria are the input parameters that the system uses to simulate. HOMER Pro is a thorough financial and environmental analysis program that evaluates carbon emissions and provides information on a project’s financial viability by calculating payback periods, capital costs, and operational expenses.

**Fig. 10:**
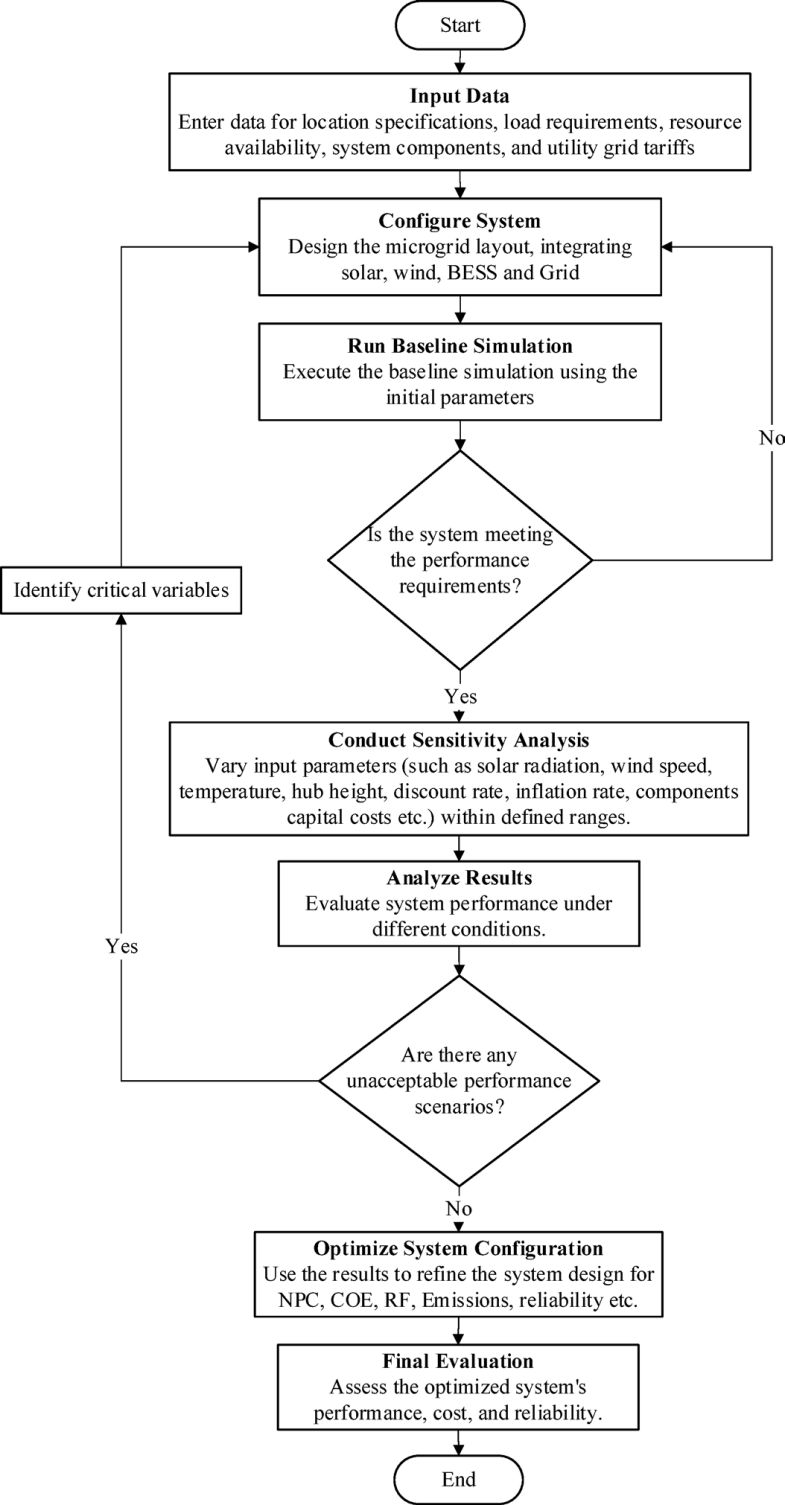
Methodology flowchart of the proposed work.

It should be noted that HOMER Pro internally applies standardized and validated component models, dispatch strategies, and degradation assumptions. The mathematical formulations presented here are used to explain the underlying physical behavior, while final system performance metrics are computed using HOMER’s integrated optimization engine. The workflow, presented in Fig. 10, begins with dataset preparation and system configuration. HOMER then performs baseline simulations, evaluates performance under varying conditions, and conducts sensitivity analyses to identify critical influencing parameters^128^. This iterative process helps determine the least-cost configuration with maximum reliability and minimal emissions.

HOMER Pro also accounts for life-cycle costs, battery degradation, and environmental impacts, ensuring that selected systems are not only economical but also sustainable over their operational lifetime^129–131^.

**Fig. 11:**
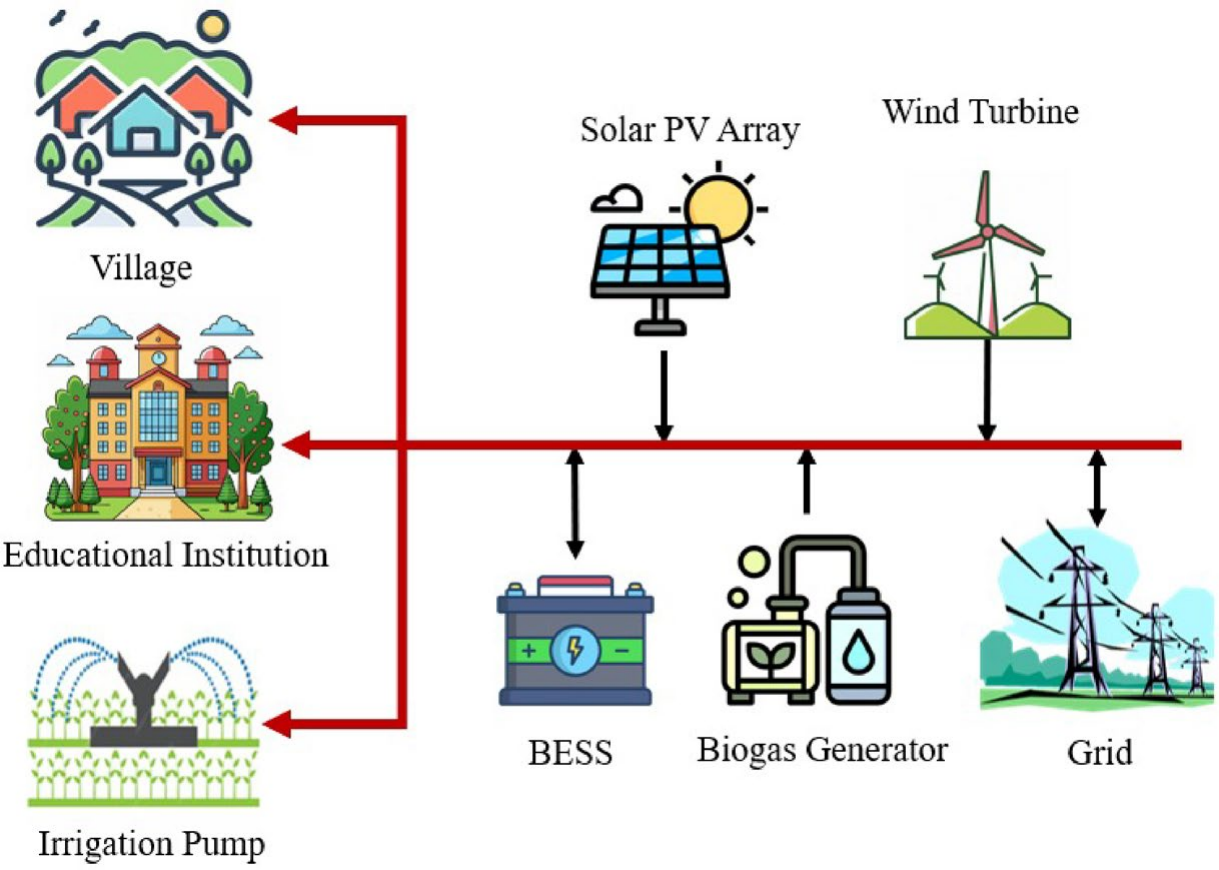
HRES schematic diagram of the proposed microgrid.

HRES integrates all the various renewable energy sources, including WT, solar PV, BioGen, BESS, and grid power, in the pursuit of constant energy supply with increased efficiency^132^. The Fig. 11 shows the schematic of the hybrid renewable energy system (HRES) used in this study, incorporating solar PV, wind turbines, a biogas generator, BESS, and grid power. This integrated system is designed to provide reliable electricity to households, educational institutions, and irrigation pumps, improving energy access, reducing outages, and enhancing long-term sustainability in the study area^133,134^.

**Fig. 12:**
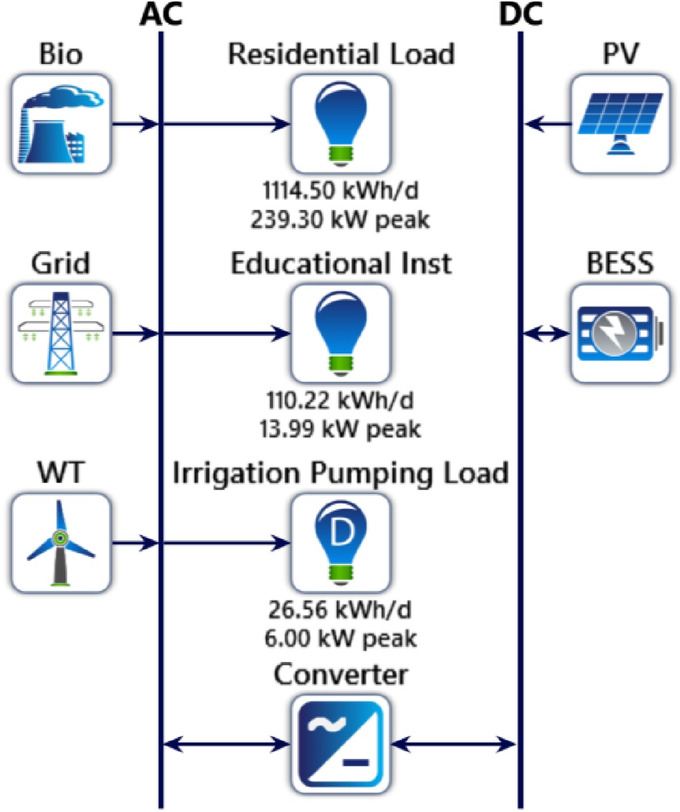
HOMER Pro simulation schematic for the proposed microgrid.

Figure 12 presents an integrated AC–DC hybrid microgrid architecture combining multiple energy sources and load types. On the AC bus, biogas, grid supply, and a wind turbine feed three major demand sectors: a residential load of 1114.50 kWh/day with a 239.30 kW peak, an educational institution consuming 110.22 kWh/day with a 13.99 kW peak, and an irrigation pumping load of 26.56 kWh/day with a 6.00 kW peak. A bidirectional converter links the AC and DC networks. On the DC side, photovoltaic (PV) generation and a battery energy storage system (BESS) ensure power balancing, reliability, and enhanced operational flexibility within the microgrid.

### Dynamic stability analysis

To evaluate the voltage and frequency behavior of the proposed hybrid microgrid, a sensitivity-based linear dynamic model was developed in MATLAB to provide a first-order, screening-level assessment of system stability at the point of common coupling (PCC). The model characterized small-signal deviations in voltage and frequency as linear functions of active and reactive power imbalances, represented through proportional sensitivity gains. Frequency deviation was described using a first-order dynamic relation governed by a frequency sensitivity coefficient (k_f_) and an associated time constant, while voltage deviation was modeled in an analogous manner using a voltage sensitivity coefficient (k_v_). These parameters were chosen to represent a moderately stiff rural grid, consistent with inverter-dominated microgrids reported in previous studies. Under step changes in load and renewable generation, typical recovery times of approximately 1–1.5 s were obtained, indicating stable operation within grid-code voltage and frequency limits for the modeled scenarios. This assessment was intended as an initial screening tool for voltage–frequency stability rather than a detailed electromagnetic transient or inverter-level validation. Nonetheless, the results provided useful insight into the dynamic response of the proposed configuration and its capability to maintain acceptable operating limits under expected operating conditions. For completeness, the model assumed droop-based frequency control, with proportional voltage–frequency sensitivity coefficients (k_f_ and k_v_) used to emulate inverter behavior. The rural grid was treated as moderately stiff, corresponding to a short-circuit ratio (SCR) of approximately 3, which is representative of typical rural distribution networks. Disturbance scenarios consisted of step changes in load and renewable generation to evaluate the system response. No severe faults or islanding events were simulated, as the objective of this screening-level analysis was to illustrate overall stability trends rather than to capture detailed electromagnetic transient behavior.

### Techno-economic specifications

The techno-economic parameters include the rated capacities, capital and replacement costs and annual O&M costs of the main components of system (PV array system, WT, power converter) are presented in Table 2. A cost effective 10 kW small wind turbine for the micro-series applications is a generic horizontal-axis wind turbine (HAWT) that presents superior features such as higher capacity and efficiency when compared to vertical-axis turbines, but still provides affordable low-maintenance decentralized energy generation^135^. The system includes PV modules rated at 1 kW, a 10 kW WT, a 1 kW converter, a 1 kW BioGen, and a 5.12 kWh BESS. Capital and replacement costs are detailed for each component, with PV and WT systems having the highest initial investment per kW. The variables are needed to determine system performance, estimate life cycle cost, and conduct financial analysis for ensuring the viability and sustainability of the hybrid renewable energy system.

**Table 2:** Techno-Economical summary of the components.

Parameter	PV	WT	Converter	BioGen	BESS
Rated Capacity	1 kW	10 kW	1 kW	1 kW	5.12 kWh
Capital Cost ($)	300/kW	3000/unit	118/kW	85/kW	950
Replacement Cost ($)	300/kW	3000/unit	100/kW	70/kW	950
O&M Cost ($/yr)	10/kW	50/unit	10/kW	0.07/kW	1
References	^136^	^137^	^138^	^139^	^140^

## Results and discussion

HOMER Pro is an industry-standard optimization and simulation software used for designing hybrid renewable energy systems. It evaluates thousands of possible configurations by performing techno-economic analysis, simulating hourly system performance, and identifying the most cost-effective and reliable combination of components. Through iterative optimization, HOMER Pro eliminates infeasible designs, examines component interactions, and quantifies key metrics such as renewable fraction, unmet load, emissions, and lifecycle cost. This makes it highly suitable for assessing microgrid configurations under various resource and load conditions. Among the 811 solutions simulated, 380 were feasible, while 431 were infeasible due to capacity shortage constraints. In addition, 205 solutions were omitted prior to full evaluation: 0 due to infeasibility, 123 because they lacked a necessary converter, and 50 for having an unnecessary converter.

Table 3 summarizes the eight case studies analyzed. The systems included combinations such as PV–WT–BioGen–BESS–Grid–Converter (Case I), PV–WT–BESS–Grid–Converter (Case II), WT–BioGen–BESS–Grid–Converter (Case III), and others. These configurations were evaluated over a 25-year period. Optimal systems—particularly those combining PV, wind, BioGen, and BESS—achieved renewable fractions of up to 88.2%, improving reliability and reducing CO₂ emissions. Systems without full hybridization were more cost-effective but showed lower renewable penetration and higher unmet load, illustrating the importance of balanced component selection and converter compatibility in microgrid design.

**Table 3:** Summary of the different case study.

Components	Case Study
BESS-Grid-Converter	Base Case
PV-WT-BioGen-BESS-Grid-Converter	Case-I
PV-WT- BESS-Grid-Converter	Case-II
WT-BioGen-BESS-Grid-Converter	Case-III
PV- BioGen-BESS-Grid-Converter	Case-IV
WT- BESS-Grid-Converter	Case-V
PV- BESS-Grid-Converter	Case-VI
BioGen-BESS-Grid-Converter	Case-VII

### Techno economic analysis

This techno-economic assessment investigates the practicality, cost efficiency, and sustainability of different hybrid energy systems designed for residential and commercial applications. By combining grid connectivity, energy storage, and renewable power sources, the analysis aims to ensure reliable electricity supply while lowering operational expenses and promoting cleaner energy alternatives suitable for diverse user needs.

The accompanying Table 4 summarizes the performance of several case studies, comparing them across lifecycle cost, energy pricing, operational expenditures, initial investment, renewable penetration, energy exchanged with the grid, and emission levels. The results highlight that systems integrating multiple renewable sources with storage generally achieve lower long-term costs and reduced environmental impact, whereas configurations with limited renewable input exhibit higher dependence on grid power and increased operational and environmental burdens.

**Table 4:** Comparative techno-economic and environmental indicators for hybrid microgrid configurations.

Case Study	NPC ($)	COE ($)	Operating cost ($/yr)	Initial capital ($)	Ren Frac (%)	Energy Purchased (kWh)	Energy Sold (kWh)	CO_2_ & CO (kg/yr)	SO_2_ & NO_x_ (kg/yr)
Base Case	690144.8	0.113	44796.02	89522.97	0	460199.3	0	290,846 & 0	1,261 & 617
Case-I	206840.7	0.0207	4088.05	152028.4	88.19	88242.07	290268.3	55,806 & 0.406	242 & 118
Case-II	224049.2	0.0225	3928.48	171376.5	79.43	152692.5	285724.3	96,502 & 0	418 & 205
Case-III	314,464	0.0477	19560.2	52202.24	71.65	139273.2	34709.57	88,081 & 0.665	382 & 187
Case-IV	372605.3	0.0447	16736.21	148207.3	76.95	143393.3	165702.7	90,689 & 0.718	393 & 193
Case-V	396791.6	0.0606	23558.26	80924.16	49.17	248112.2	31620.42	156,807	680
Case-VI	420511.8	0.0508	18422.27	173507.3	57.98	259,632	161541.6	164,087	711
Case-VII	578920.5	0.0944	39085.81	54860.67	55.52	203382.3	816.84	128,675	557

The capital, O&M, and replacement costs used in this study are based on recent literature and commercially available global estimates, reflecting typical values for small-scale hybrid systems. While site-specific costs in Bangladesh may vary due to local labor, supply-chain conditions, and import tariffs, these values provide a reasonable baseline for techno-economic comparison. Future work will incorporate detailed Bangladesh-specific market data for refined analysis.

**Fig. 13:**
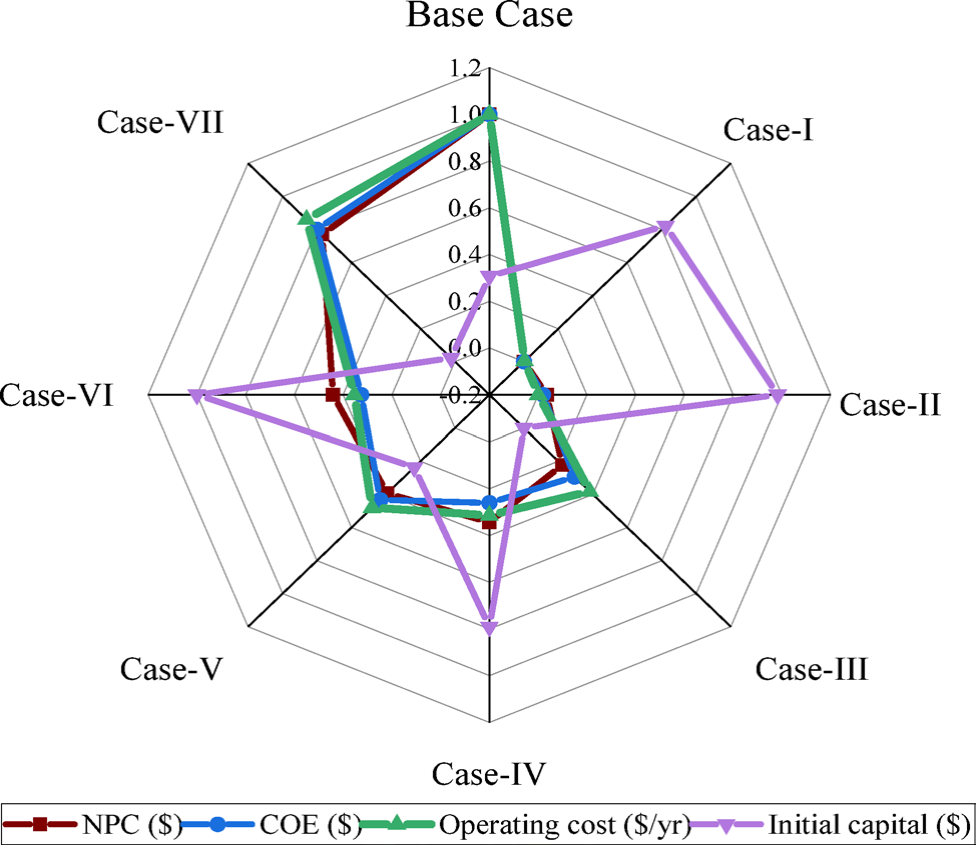
Normalized techno-economic indicators for all case studies.

Figure 13 presents a comparative visualization of the normalized techno-economic indicators-namely, NPC, COE, operating cost, and initial capital-for all case studies. Normalization was performed by using a 0–1 linear scale to enable direct multi-criteria comparison. Correspondingly, the Base Case indicates the highest NPC (690,144.8 $; normalized = 1.0) and COE (0.1128 $/kWh; normalized = 1.0), which is indicative of its much higher cost burden. Case-I indicates a minimum COE of 0.02066 $/kWh (normalized ≈ 0.0204) and one of the minimum operating costs of 4,088.05 $/yr (normalized ≈ 0.0039). Case-II and Case-III are similar in mid-range patterns with a normalized NPC value of 0.0356 and 0.2268, respectively. Case-V and Case-VI have shown a moderate value of NPC and COE. Case-VII shows a relatively higher cost burden with NPC value of 578,920.5 $ (normalized = 0.770) and operating cost of 39,085.81 $/yr (normalized = 0.860). The radar plot has indicated the superiority in terms of the cost efficiency of Case-I, while the Base Case and Case-VII represent high financial burdens.

**Fig. 14:**
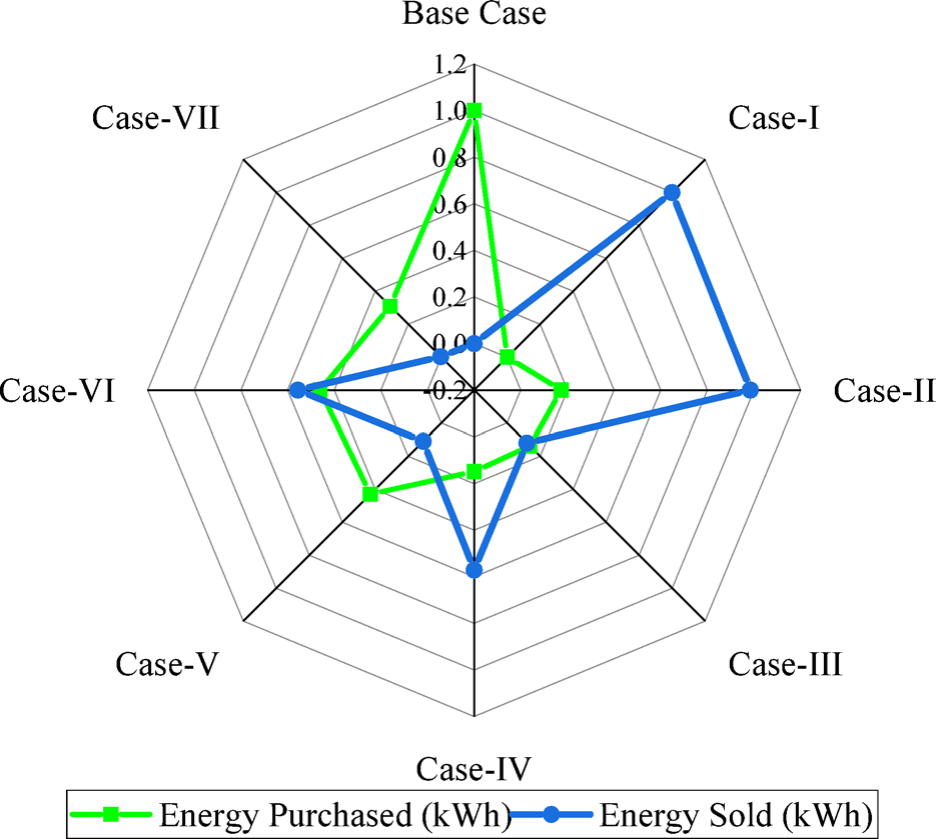
Comparative normalized energy exchange profile across all case studies.

Figure 14 shows the normalized values of energy purchased and energy sold for all case studies to enable direct comparison by a 0–1 scaling method. The Base Case has the highest grid dependence, purchasing energy at 460,199.3 kWh (normalized = 1.0) with no energy sold, 0 kWh (normalized = 0). This is a fully grid-importing profile. Case-I has the highest export performance: it sells 290,268.3 kWh (normalized = 1.0) while importing only 88,242.07 kWh (normalized ≈ 0.1917). Case-II also has very good export capability: it sells 285,724.3 kWh (normalized ≈ 0.9844) with moderate purchase, 152,692.5 kWh (normalized ≈ 0.1733). Cases-III through VII show various mixed-import behaviors, with their normalized energy-purchased value varying from 0.1372 to 0.4608, while their sales are substantially lower than that of Case-I and Case-II. Case-VII has very low export, 816.84 kWh (normalized ≈ 0.0028), despite a moderate purchase, 203,382.3 kWh (normalized ≈ 0.3096). The radar chart emphasizes the superior energy-exporting performance of Case-I and Case-II, while the strong grid dependence of the Base Case and Case-VII are also reflected.

**Fig. 15:**
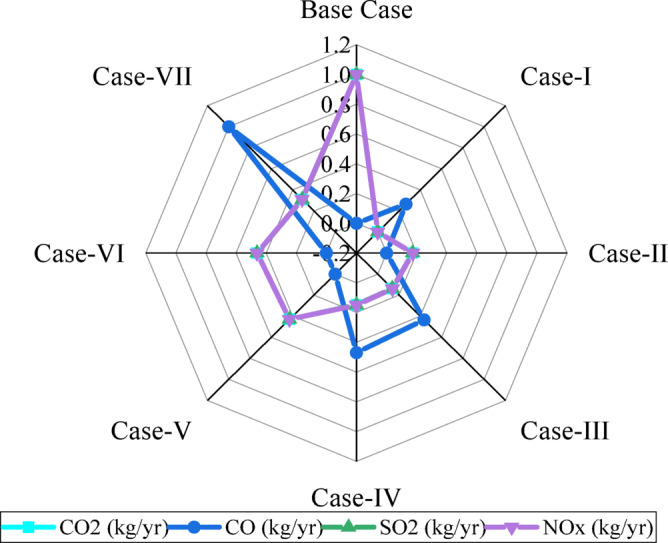
Comparative radar plot of normalized atmospheric emissions for evaluated energy configurations.

Figure 15 illustrates the normalized atmospheric emissions of CO₂, CO, SO₂, and NOₓ for all case studies on a 0–1 scale. The Base Case represents the highest emissions in each category, including CO₂ = 290,846 kg/yr (normalized = 1.0), SO₂ = 1,261 kg/yr (1.0), and NOₓ = 617 kg/yr (1.0), while yielding zero CO. Case-I presents much lower emissions, including CO₂ = 55,806 kg/yr (0.192), SO₂ = 242 kg/yr (0.192), and NOₓ = 118 kg/yr (0.192), whereas CO remains very small at 0.406 kg/yr (normalized = 0.265). Case-II and Case-III result in moderate emissions, while their normalized CO₂ values are 0.173 and 0.138, respectively. Case-V and Case-VI produce higher pollutant levels compared to other renewable-rich cases, while Case-VII shows higher CO emissions at 1.53 kg/yr (normalized = 1.0). The radar pattern of the plot reveals significant emissions reduction in the hybrid renewable configurations compared to the Base Case.

While Fig. 15 reports CO₂, CO, SO₂, and NOₓ emissions arising from grid electricity and biogas-based generation, this analysis does not account for lifecycle emissions associated with the manufacturing, transportation, installation, and end-of-life disposal of PV modules, wind turbines, and battery systems. Inclusion of these upstream and downstream emissions would enable a more comprehensive environmental assessment and is therefore identified as an important direction for future research to further strengthen the sustainability evaluation of rural hybrid microgrids.

**Fig. 16:**
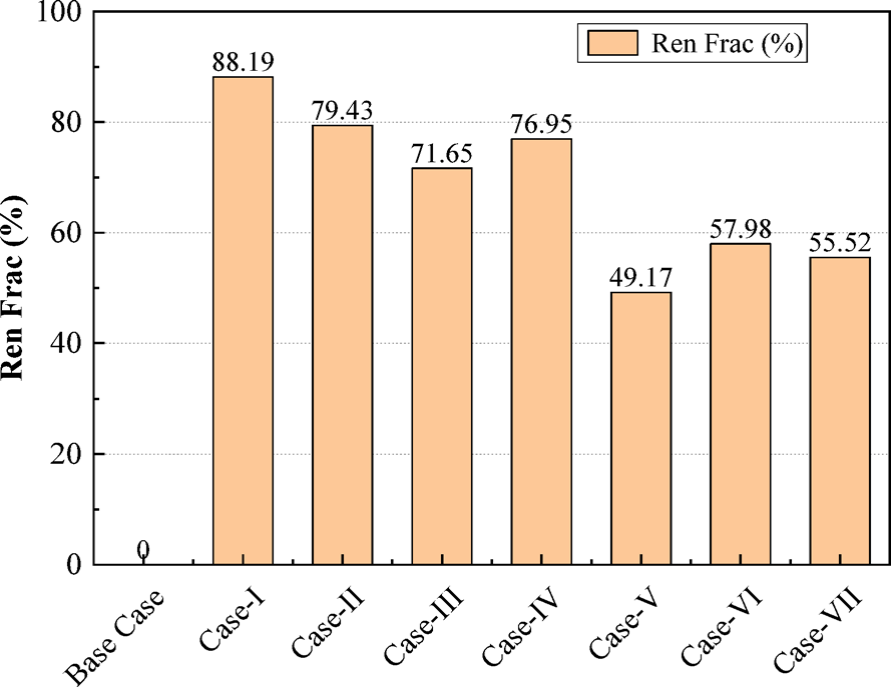
Renewable energy contribution under optimized microgrid scenarios.

Figure 16 illustrates the renewable energy fraction achieved across all case studies, highlighting the contribution of renewable sources to the overall energy supply. The Base Case records 0%, indicating complete dependence on non-renewable or grid-based electricity. Case-I demonstrates the highest renewable penetration at 88.19%, followed by Case-II at 79.43% and Case-III at 71.65%, reflecting the strong impact of hybrid PV–wind–BioGen systems supported by storage. Case-IV achieves 76.95%, performing comparably to Case-III. Case-V, containing fewer renewable components, shows a significantly lower renewable fraction of 49.17%. Case-VI and Case-VII exhibit moderate renewable integration at 57.98% and 55.52%, respectively. Overall, the figure indicates that systems with diverse renewable sources and adequate storage achieve substantially higher renewable fractions, while configurations with limited renewable input or dependence on dispatchable generators exhibit lower penetration levels.

### Optimum case

**Fig. 17:**
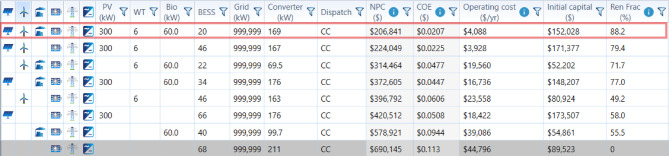
Optimized system configurations and techno-economic results from HOMER Pro.

The best configuration is Case I, as it achieves the best balance among the cases regarding economic performance, integration of renewables, and environmental benefits. It records the lowest NPC ($206,840.7) and lowest COE ($0.0207), hence indicating excellent long-term affordability. Additionally, in renewable-rich cases, it has the lowest operating cost at $4,088/yr. The initial capital requirement is more than that of some alternatives, but the saving on long-term costs substantially outweighs the front investment. This Fig. 17 presents the optimized component sizing and corresponding techno-economic performance of the evaluated hybrid microgrid configurations obtained from HOMER Pro. It reports the final installed capacities of PV, wind turbines, biogas generator, battery energy storage, grid and converter ratings, along with dispatch strategy, NPC, COE, operating cost, initial capital, and renewable fraction. The figure highlights the optimal configuration (Case I), which achieves the lowest NPC and COE while maintaining a high renewable penetration.

**Fig. 18:**
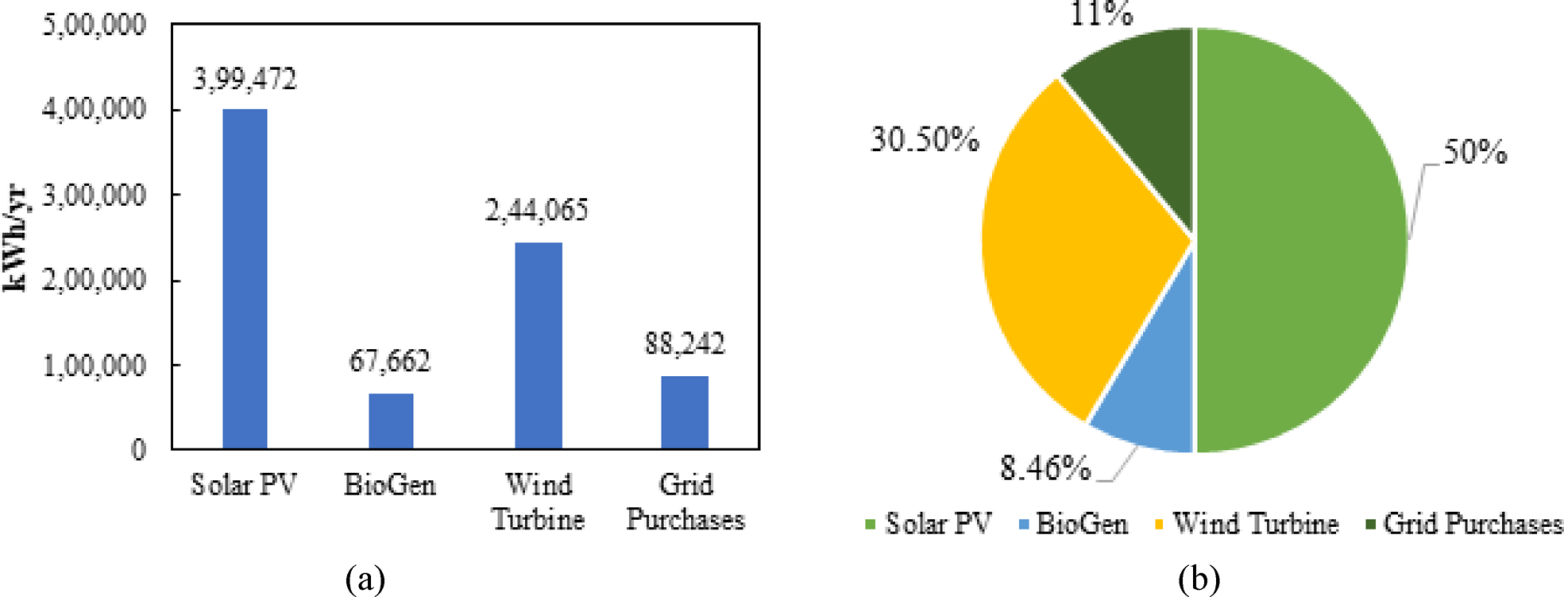
Annual energy contribution of the proposed microgrid: **(a)** yearly energy amounts and **(b)** percentage share of each source.

Figure 18 illustrates the annual energy contribution of each source in the optimum hybrid configuration. Figure 18(a) shows that Solar PV delivers the highest output at 399,472 kWh/yr, followed by wind turbines at 244,065 kWh/yr. BioGen contributes 67,662 kWh/yr, while grid purchases supply 88,242 kWh/yr. Figure 18(b) presents the proportional energy share, where Solar PV accounts for 50%, wind turbines 30.50%, BioGen 8.46%, and grid purchases 11%. Overall, the system primarily relies on renewable sources with limited dependence on the grid.

Case I reaches the highest value of Renewable Fraction (88.19%), reflecting better dependence on PV–WT–BioGen–BESS synergies from the point of sustainability. This high renewable penetration reduces grid purchases and drives significant reduction in emissions, showing the lowest CO₂ and pollutant outputs among all the renewable-integrated cases. In radar plot comparisons, Case I dominates consistently across key axes like NPC, COE, renewable fraction, emissions, and grid reliance to form the largest and most balanced polygon. Other cases suffer from either higher costs, such as Case VI and VII, or lower renewable contribution, such as Case V and VII, and elevated emissions despite moderate renewable input, as in Case-III and IV. Collectively, these indicators confirm that Case I provides the most cost-effective, sustainable, and operationally efficient solution.

**Fig. 19:**
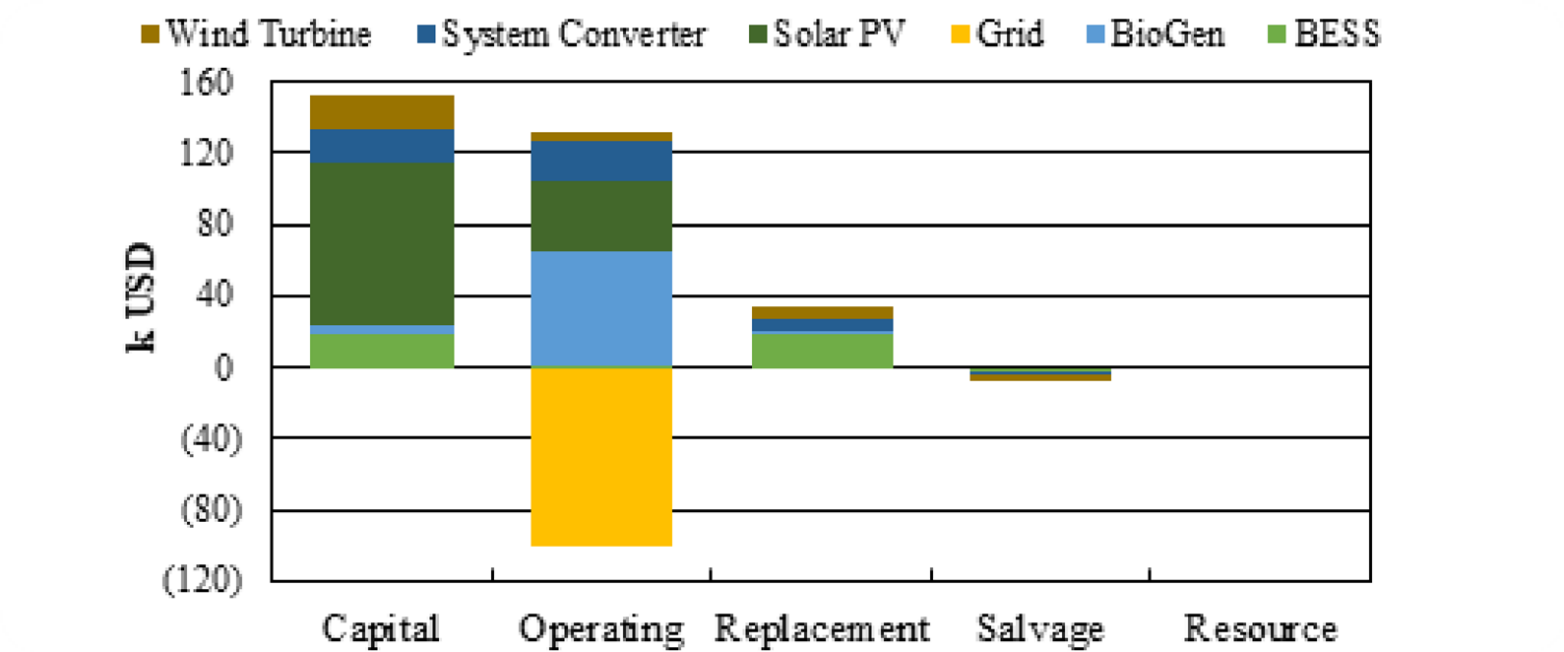
NPC contribution by individual components in the optimal configuration.

The cost breakdown in Fig. 19 shows how each component contributes to the total system NPC of $206,841. Solar PV represents the largest share, with a capital cost of $90,000 and operating cost of $40,224, totaling $130,224, making it the primary investment driver. The system converter is the second-largest contributor at $48,658, including $19,928 capital and $22,644 operating cost. BioGen adds a significant $70,069, driven mainly by its high operating cost of $63,972, despite a modest capital cost of $5,100. BESS contributes $34,404, combining $19,000 capital and $17,625 replacement cost, partly offset by salvage. Wind turbine costs total $24,650, with moderate capital and replacement expenses. The grid provides the only negative contribution at –$101,164, reflecting revenue from excess energy sales. Overall, the figure visually highlights PV, converter, and BioGen as the major cost components shaping the system’s total NPC.

**Fig. 20:**
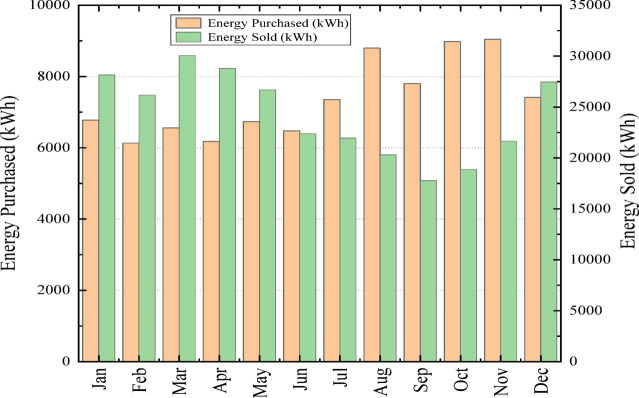
Monthly energy purchased and sold for the optimal case I configuration.

Figure 20 presents a monthly comparison of energy purchased from and sold to the grid for the optimal Case I hybrid system. Across the year, energy sold consistently exceeds energy purchased, demonstrating strong renewable generation and effective integration of PV, wind, and BioGen resources. Monthly energy purchased remains relatively stable, ranging from 6,133 kWh in February to a maximum of 9,042 kWh in November, resulting in a total annual purchase of 88,242 kWh. In contrast, energy sold shows pronounced seasonal variability, with the highest export occurring in March (30,053 kWh) and the lowest in September (17,780 kWh), contributing to a substantial annual export of 290,268 kWh. This surplus highlights Case I’s capability to not only meet local demand but also supply excess clean energy to the grid. Overall, the figure emphasizes the system’s strong renewable penetration and favorable energy balance throughout the year.

**Fig. 21:**
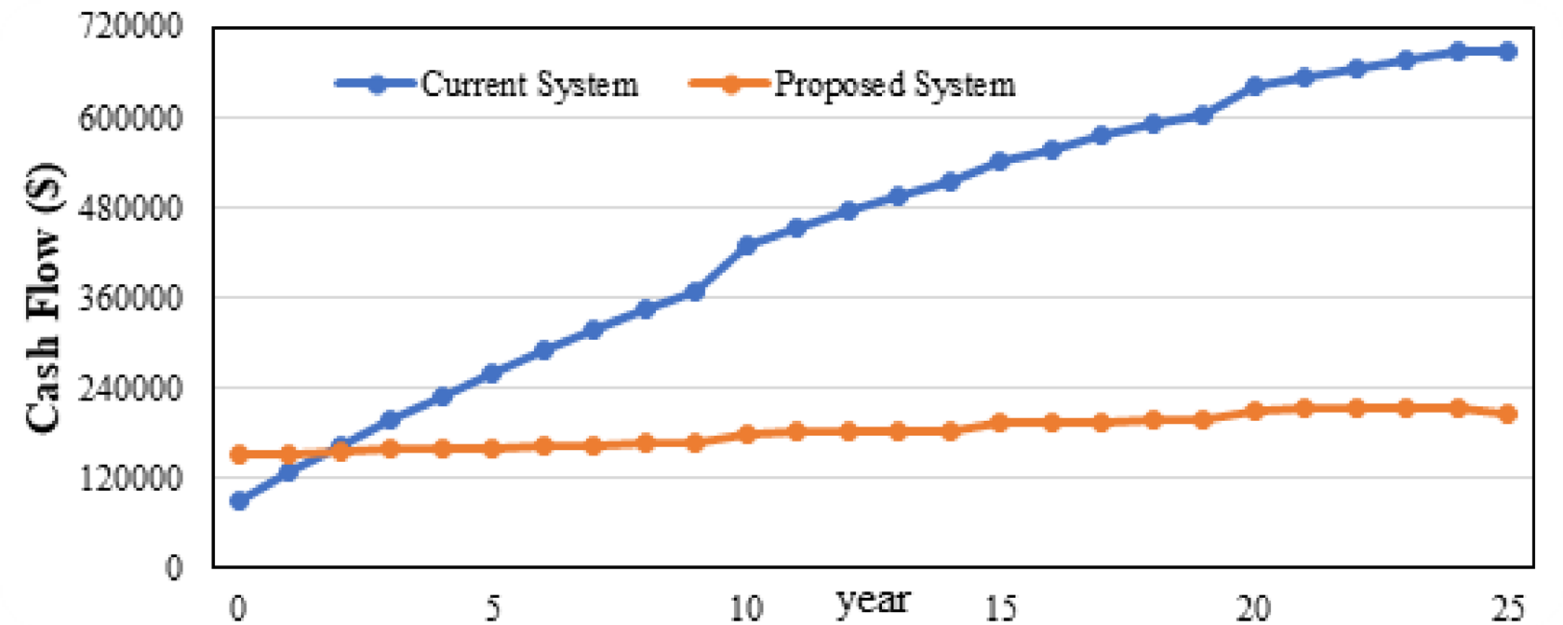
Cumulative cash flow over project lifetime.

Figure 21 highlights the substantial economic advantage of the proposed hybrid energy system relative to the base system. In the plotted trend, the current (base) system shows a steep cumulative cash-flow increase driven by high operating expenditures, reflected in its NPC) of $690,145 and annual OPEX of $44,796. Conversely, the proposed system maintains a flatter cash-flow trajectory due to markedly lower lifecycle costs, supported by a reduced NPC of $206,841 and minimal OPEX of $4,088 despite a higher initial capital investment (CAPEX $152,028 vs. $89,523). Economic performance indicators confirm the superiority of the proposed configuration: simple payback of 1.64 years, discounted payback of 1.77 years, and a high IRR of 61.4%, demonstrating rapid recovery of investment and strong long-term financial viability. The system also achieves a much lower LCOE ($0.0207/kWh) and significantly reduces emissions, cutting CO₂ from 290,846 to 55,806 kg/yr, making it both economically and environmentally optimal.

**Fig. 22:**
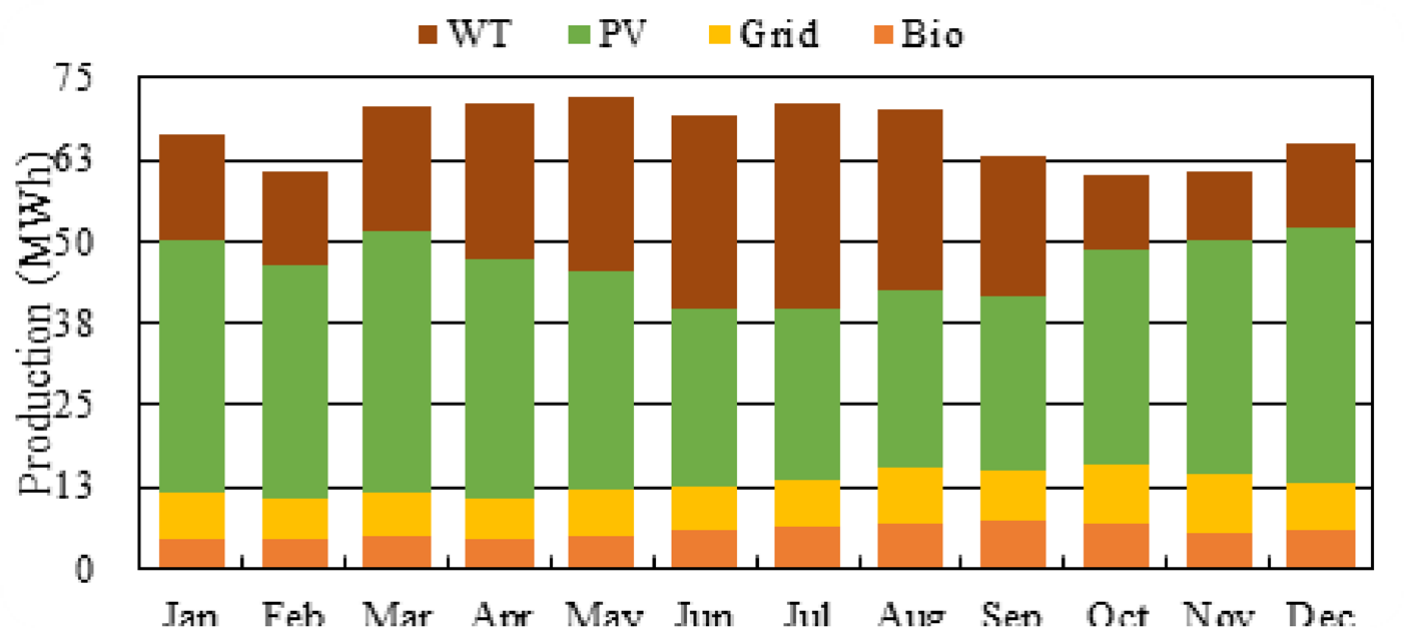
Monthly renewable and grid energy production (MWh).

Figure 22 depicts the monthly electricity generation mix of the proposed hybrid microgrid, which has to meet 2046 kWh/day with a 250 kW peak demand. The total microgrid demand of 2046 kWh/day and 250 kW peak represents the combined residential (100 households), educational, and irrigation loads modeled in Sect. “Residential load profile”–“Deferrable load profile”. The system has four contributors: wind turbines, photovoltaic panels, grid purchases, and biogas generators. The contribution of PV ranges from 33.50 to 40.18 MWh/month, peaking during March with 40.18 MWh because of better solar irradiance. The second largest contribution is that of wind generation, which ranges between 11.58 and 31.36 MWh and peaks in July, illustrating seasonal variations in the wind resource. BioGen produces a pretty fair amount of energy over the year, ranging between 4.35 and 7.10 MWh, which would be essential to ensure dispatchability during those months when renewable energy resources are scarce. Grid purchases are only 6.13 to 9.05 MWh, showing strong renewable penetration with reduced dependency on external supply. Overall, the above figure depicts the contribution of the proposed system, dominated by PV (≈ 55–60%) and WT (≈ 30–35%). In addition, BioGen and limited amounts of grid energy secure reliability. A hybrid configuration balanced in this way can serve the load under study with high renewable integration and good operational stability.

**Fig. 23:**
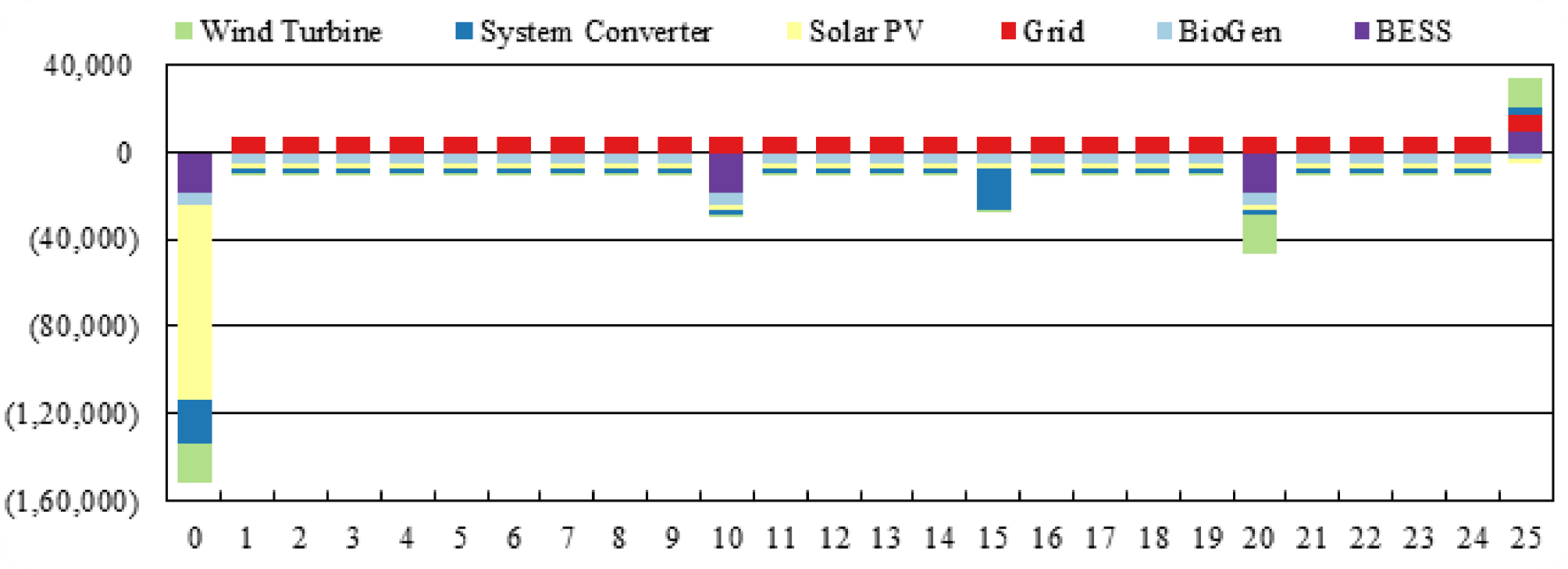
Annualized cash flow distribution across system components for the proposed microgrid.

Figure 23 presents the annualized cash flow contributions of each component in the proposed microgrid for a project lifetime of 25 years. A positive bar represents cost outflows, mostly dominated by grid charges, which are shown to be around $+8,000 to $+10,000 every year; and the negative bars reflect capital, operating, and replacement costs. Solar PV has the highest initial cost burden of nearly –$140,000 at Year 0, followed by BESS and system converter at about –$120,000 and –$60,000, respectively. Wind turbines and BioGen only have a relatively small negative amount between –$20,000 and –$40,000 during their scheduled replacement years. Observe the sharp declines in mid-life years around Year 10 and Year 20 due to scheduled replacement costs of the PV, BESS, and wind components. Minor salvage values in Year 25 generate a small positive cash inflow. This graph illustrates that while the microgrid requires high upfront investments mainly from PV and storage, the long-term operational costs remain low and support good economic viability.

**Fig. 24:**
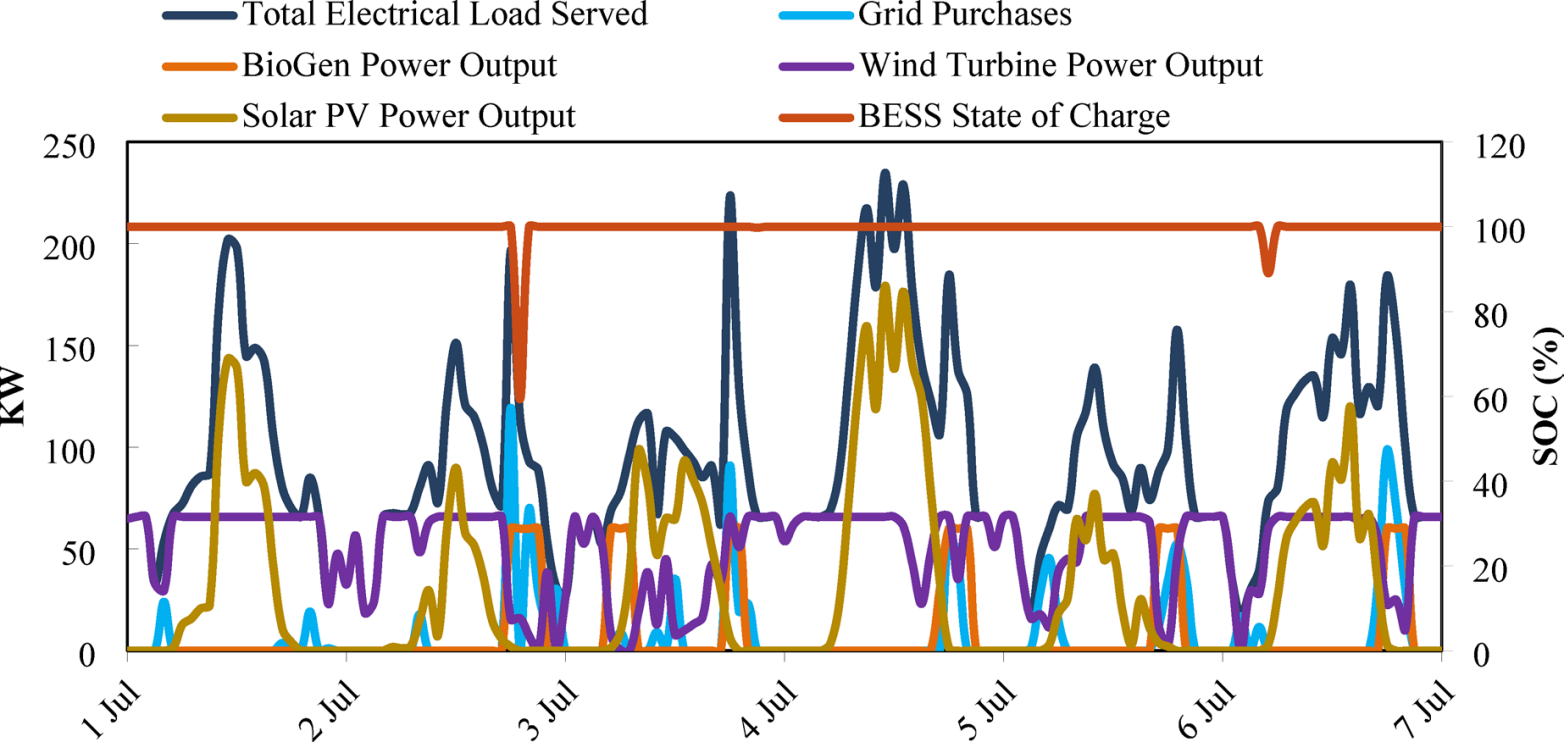
Temporal variation of generation, storage, and load in Case I microgrid.

Figure 24 presents the hybrid microgrid performance from 1 to 7 July, showing power generation, load served, and BESS SOC. The Total Electrical Load Served fluctuates between 40 and 220 kW, reaching peaks above 210 kW on 4–5 July. Solar PV output varies from 0 to ~ 160 kW, following the daily solar cycle. Wind turbine generation ranges roughly between 10 and 70 kW, reflecting variable wind conditions. BioGen operates as a dispatchable backup source with variable output up to its rated capacity of 60 kW., with brief dips on 3 and 6 July. Grid purchases occur mainly when renewable output falls below 50 kW. The BESS maintains a high SOC of 95–100%, indicating that stored energy is seldom discharged. Overall, the figure demonstrates coordinated interactions among renewable sources, BioGen, and grid support to meet the system load.

**Fig. 25:**
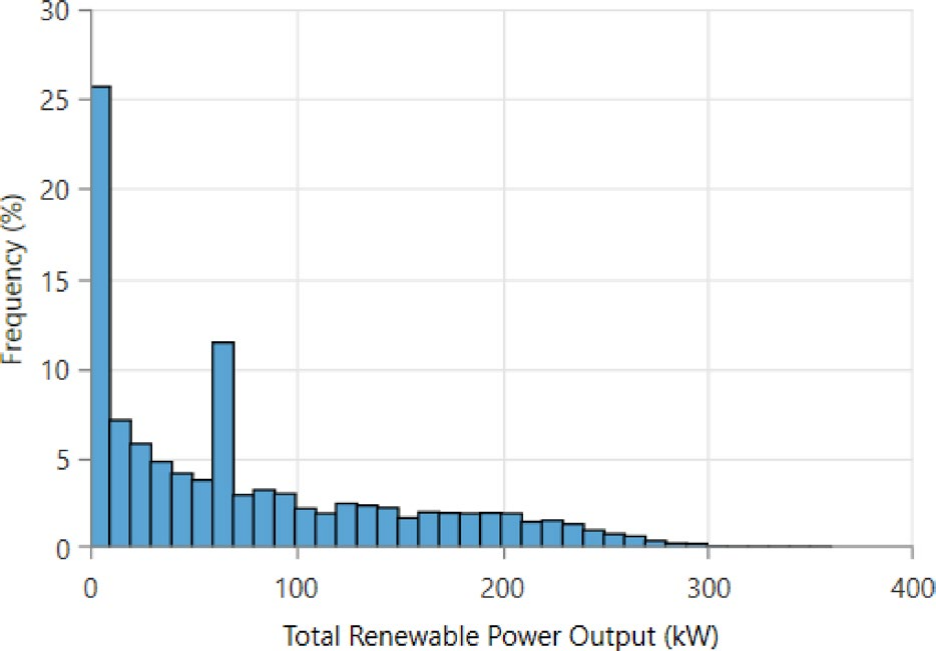
Histogram of total renewable power output.

Figure 25 illustrates the frequency distribution of total renewable power output from the hybrid system. Most occurrences fall within the 0–20 kW range, accounting for over 25% of the time, indicating frequent low-generation periods. A secondary cluster appears around 60–80 kW with roughly 12% frequency. Higher outputs above 150 kW occur less than 3% of the time, and outputs exceeding 300 kW are rare. The distribution highlights the intermittent nature of combined solar-wind renewable generation and the need for complementary dispatchable or storage resources.

**Fig. 26:**
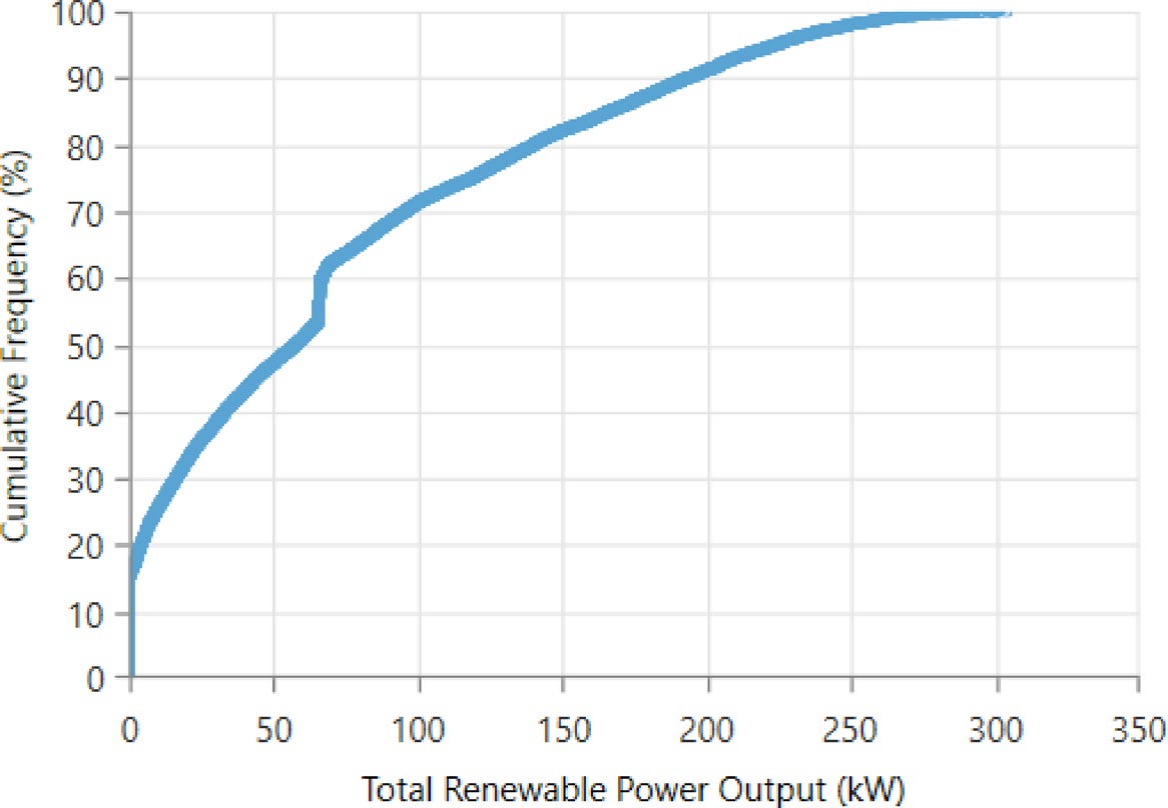
Cumulative Distribution Function (CDF) of total renewable power output.

Figure 26 presents the cumulative distribution of total renewable power output from the hybrid system. Around 20% of the time, renewable generation is below 20 kW, reflecting frequent low-output conditions. The curve shows that 50% of all renewable output values fall below approximately 70 kW, while 80% remain under 150 kW. High-generation events above 250 kW occur less than 5% of the time, and outputs approach 300 kW only in rare peak conditions. The CDF highlights the variable and skewed nature of renewable generation, emphasizing the need for storage and dispatchable backup to ensure system reliability.

The comprehensive techno-economic and environmental assessment clearly shows that Case I is the most optimal microgrid configuration among all the evaluated scenarios. This hybrid system comprises PV, wind turbine, BioGen, BESS, converter, and grid support, achieves the lowest NPC of $206,841 and minimum COE of $0.0207/kWh, offering huge cost reductions compared to the base system (NPC: $690,145; COE: $0.113/kWh). Case I has an exceptionally low operating cost ($4,088/yr) with high renewable penetration of 88.19%, thus hugely reducing grid dependency. Environmental performance is similarly impressive: CO₂ emissions drop from 290,846 kg/yr in the base case to just 55,806 kg/yr, while CO, SO₂, and NOₓ emissions are also reduced substantially. Normalized radar plots reflect Case I as the top performer consistently for economic, environmental, and operational dimensions. Monthly energy flow analysis confirms stable operation, with an additional 290,268 kWh/yr sold back to the grid, further enhancing economic returns.

Overall, Case I presents the most balanced solution that combines high integration of renewables, excellent economic savings, reduced emissions, and strong operational reliability. Consequently, these results confirm its appropriateness to become the optimal configuration for the sustainable deployment of microgrids.

### Sensitivity analysis

Sensitivity analysis is conducted to evaluate the robustness of the hybrid energy system under varying environmental, economic, and infrastructural conditions. By systematically altering key input parameters, the analysis identifies which variables exert the greatest influence on system performance, cost-effectiveness, and reliability. This approach not only strengthens the validity of the model but also supports informed decision-making by highlighting parameters that require careful monitoring or optimization during system design.

Table 5 presents the full set of variables incorporated into the sensitivity analysis, each varied across five representative levels. For environmental factors, solar radiation is examined at 2.81, 3.74, 4.68, 5.61, and 6.55 kWh/m²/day, reflecting seasonal and interannual fluctuations. Temperature is varied from 15.75 °C to 36.76 °C, while wind speed ranges from 2.64 m/s to 6.17 m/s, enabling assessment of how climate-driven resource variability affects renewable energy generation. Economic parameters include inflation rates of 5.4–12.6%, nominal discount rates of 9–21%, grid power prices between 0.048 and 0.112 $/kWh, and sellback rates of 0.024–0.056 $/kWh. These values capture both favorable and adverse economic conditions impacting project cost and financial returns. Infrastructure and reliability factors include hub heights of 9.6–22.4 m, grid failure frequencies of 300–700 events/year, mean repair times of 0.6–1.4 h, and repair-time variations from 30 to 70 min. Incorporating these values allows evaluation of system resilience, operational continuity, and performance risks associated with grid instability.

**Table 5:** List of input sensitive variables with values.

Factor	Input Sensitive Variable	Values
Environmental and Resource Factors	Solar radiation (kWh/m^2^/day)	2.81, 3.74, **4.68**, 5.61, 6.55
	Temperature (ºC)	15.75, 21.01, **26.26**, 31.51, 36.76
	Wind speed (m/s)	2.64, 3.52, **4.40**, 5.28, 6.17
Economic Parameter	Inflation Rate	5.4, 7.2, **9**, 10.8, 12.6
	Nominal Discount Rate	9, 12, **15**, 18, 21
	Power Price	0.048, 0.064, **0.08**, 0.096, 0.112
	Sellback Rate	0.024, 0.032, **0.04**, 0.048, 0.056
Infrastructure and Reliability	Hub height (m)	9.6, 12.8, **16**, 19.2, 22.4
	Grid Failure Frequency	300, 400, **500**, 600, 700
	Grid Mean Repair Time	0.6, 0.8, **1**, 1.2, 1.4
	Grid Variation Repair Time	30, 40, **50**, 60, 70

The selected variation ranges reflect realistic operational uncertainties and historical data: ±40% for economic parameters corresponds to plausible fluctuations in inflation, discount rate, and energy prices, while 60–140% for environmental factors captures seasonal and interannual variability in solar irradiation and wind speed. Wind speed dominates system performance because the optimal configuration relies heavily on wind generation for renewable penetration and cost reduction. These sensitivity results inform design priorities: for example, PV sizing should consider irradiance variability, wind turbine capacity must ensure reliable generation under lower wind conditions, and grid support planning should mitigate high failure scenarios.

#### Environmental and resource factors sensitivity

**Fig. 27:**
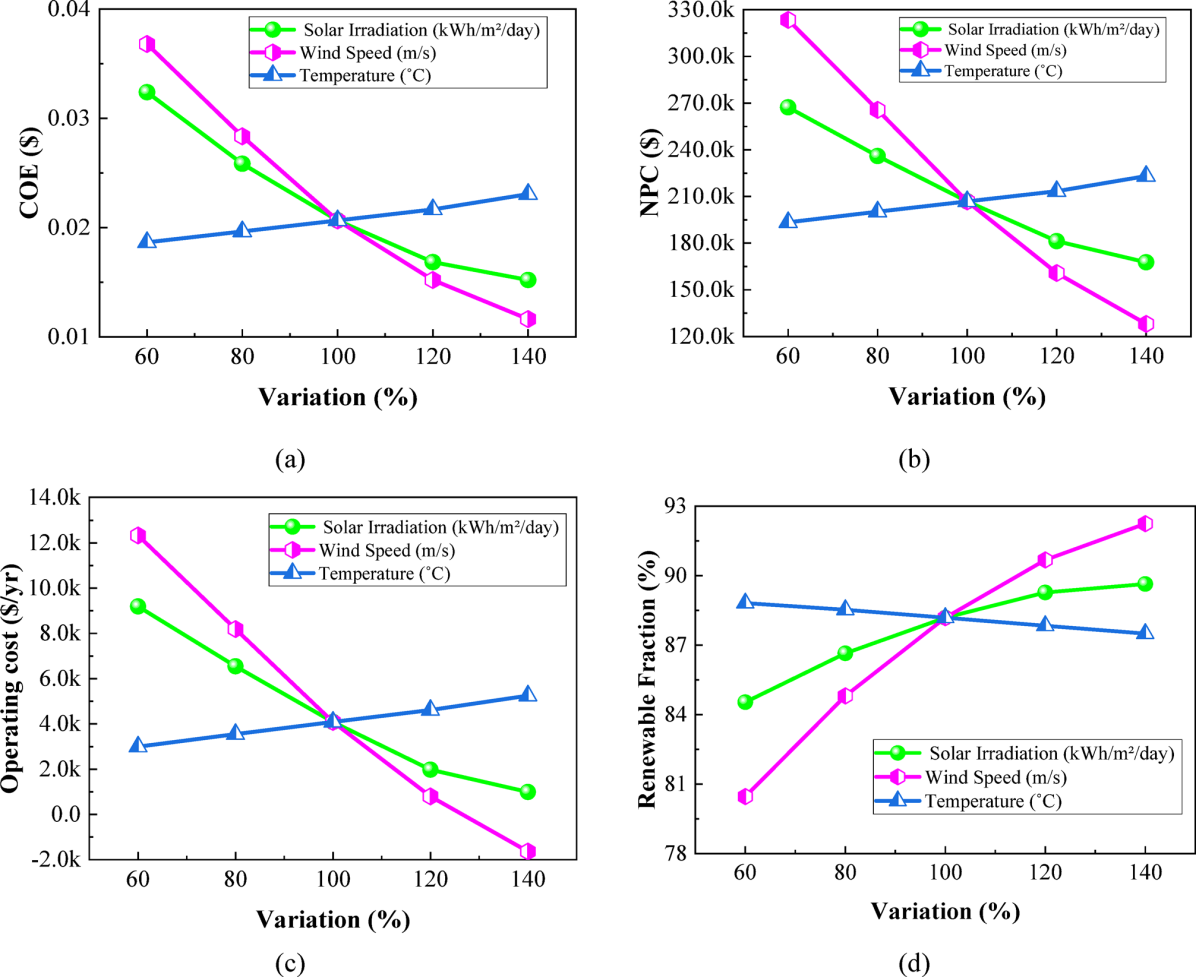
Impact of **(a)** COE, **(b)** NPC, **(c)** operating cost, and **(d)** renewable fraction on environmental and resource parameter variations.

The overall sensitivity-analysis plot—Figure 27 depicts the impact of variation in solar irradiation, wind speed, and temperature within a range from 60% to 140% of their respective baseline values on the overall performance of the system. Figure 27(a) shows that COE decreases sharply with solar irradiation from 0.03240 to 0.01521 $/kWh, while it does so even more steeply with wind speed from 0.03681 to 0.01163 $/kWh, while for temperature, a gradual increase takes place from 0.01866 to 0.02307 $/kWh. Figure 27(b) shows that NPC also experiences a corresponding drop with the increase in solar irradiation within the given range from 267,264.6 $ to 167,721.4 $ and wind speed from 323,464.4 $ to 128,113 $, while NPC increases with rising temperature from 193,399.2 $ to 223,145.6 $. As shown in Fig. 27(c), operating cost drops significantly with increased solar irradiation from 9,190.32 $/yr to 990.59 $/yr and turns negative in the case of high wind speed at − 1,639.25 $/yr, a reflection of surplus generation of wind; temperature raises operating cost from 2,993.33 $/yr to 5,250.65 $/yr. Figure 27(d) presents renewable fraction rising with solar irradiation from 84.54% to 89.64% and wind speed from 80.46% to 92.25%, but it decreases slightly with temperature from 88.82% to 87.50%. Collectively, Figs. 27(a–d) confirm that solar irradiation and wind speed exert the most beneficial influence on system economics and renewable contribution, whereas higher temperatures generally degrade performance across all metrics.

The sensitivity ranking Table 6 shows that wind speed has the strongest effect on system performance, producing the highest variations in COE, NPC, operating cost, and renewable fraction. Solar irradiation ranks second, reflecting its significant influence due to PV contribution. Temperature exhibits the lowest sensitivity, causing comparatively smaller variations across all metrics. Overall, the results indicate that renewable resource availability, especially wind, governs the system’s techno-economic responsiveness.

**Table 6:** Ranked impact of environmental and resource variations on hybrid system metrics.

Rank	Parameter	Sensitivity Level	Metrics Affected	Reason
1	Wind Speed	Very High	COE, NPC, Operating Cost, RF	COE decreases by 68.4%, NPC decreases by 60.4%, operating cost decreases by 113%, and the renewable fraction increases by 14.7%. This strong impact occurs because the system relies heavily on wind power.
2	Solar Irradiation	High	COE, NPC, Operating Cost, RF	COE decreases by 53.1%, NPC decreases by 37.2%, operating cost decreases by 89.2%, and the renewable fraction increases by 6%. This high sensitivity is due to the significant share of solar PV in the system.
3	Temperature	Low–Moderate	COE, NPC, Operating Cost, RF	COE increases by 23.7%, NPC increases by 15.4%, operating cost increases by 75.4%, and the renewable fraction decreases by 1.5%. The limited impact is because temperature affects only PV efficiency and has no influence on other generation units.

#### Economic parameter sensitivity

**Fig. 28:**
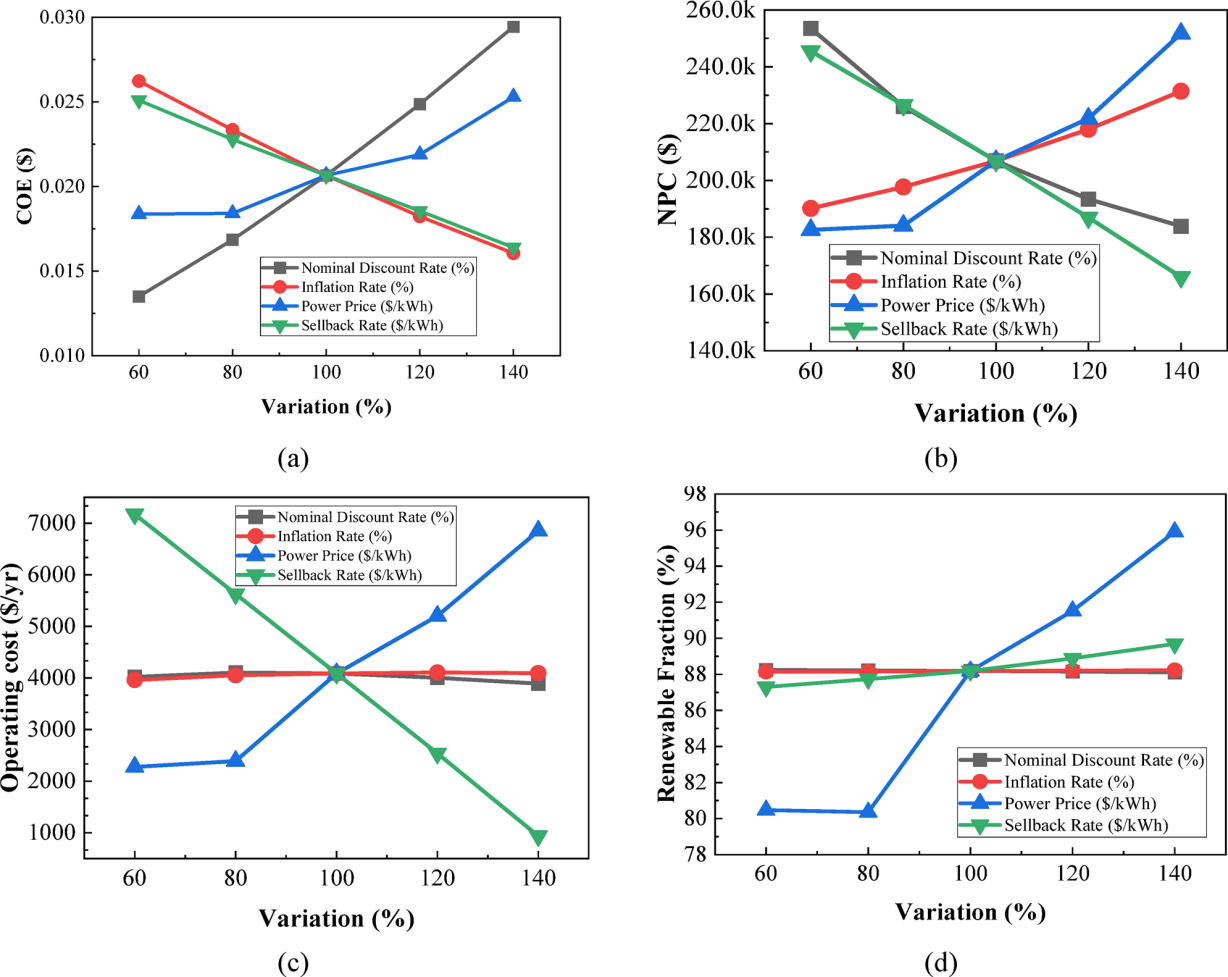
Impact of **(a)** COE, **(b)** NPC, **(c)** operating cost and **(d)** renewable fraction on economic parameter variations.

The four-panel sensitivity plot in Fig. 28 illustrates how variations of ± 40% around the base value (60–140%) in key economic parameters affect COE, NPC, operating cost, and renewable fraction. Figure 28(a) COE is most sensitive to the nominal discount rate, rising sharply from 0.0135 $ to 0.0294 $ (+ 118%) as the rate increases, while inflation reduces COE from 0.0262 $ to 0.0161 $ (–39%). Power price increases COE moderately from 0.01837 $ to 0.02529 $ (+ 38%), whereas sellback rate lowers it from 0.02509 $ to 0.01640 $ (–35%). Figure 28(b) NPC similarly shows strong sensitivity: nominal discount rate causes a decrease from 253,507 $ to 183,858 $ (–27%), whereas inflation increases NPC from 190,093 $ to 231,390 $ (+ 22%). Power price exerts the highest upward impact (+ 38%), rising from 182,540 $ to 251,679 $, while sellback rate reduces NPC strongly (–32%). Figure 28(c) Operating cost increases drastically with power price from 2276 $/yr to 6852 $/yr (+ 201%), and decreases steeply with sellback rate from 7173 $/yr to 938 $/yr (–87%). Discount rate and inflation show only mild changes (± 5%). Figure 28(d) Renewable fraction is dominantly affected by power price, increasing from 80.47% to 95.90% (+ 19%), while sellback rate varies moderately (87.30–89.69%). Nominal discount rate and inflation remain nearly constant (88.24–88.11% and 88.15–88.23%). Collectively, the four sub-figures demonstrate that nominal discount rate and power price have the strongest influence on financial indicators, while sellback rate critically impacts operating cost and NPC, and power price primarily drives renewable penetration.

The sensitivity ranking Table 7 shows that power price exerts the strongest overall influence, significantly altering COE, NPC, operating cost, and renewable fraction. Sellback rate ranks second, driven by its substantial reductions in NPC and operating cost. The nominal discount rate exhibits moderate sensitivity, strongly affecting COE and NPC through long-term financial discounting. Inflation rate shows the lowest impact, inducing comparatively smaller variations across financial indicators.

**Table 7:** Sensitivity ranking of economic parameters based on output variation.

Rank	Parameter	Sensitivity Level	Metrics Affected	Reason
1	Power Price	Very High	COE, NPC, Operating Cost, RF	Causes the largest shifts: COE (+ 38%), NPC (+ 38%), operating cost (+ 201%), renewable fraction (+ 19%).
2	Sellback Rate	High	COE, NPC, Operating Cost, RF	Strong decreases in COE (–35%), NPC (–32%), and severe drop in operating cost (–87%).
3	Nominal Discount Rate	Moderate	COE, NPC	COE increases by + 118% and NPC decreases by − 27%; affects long-term financing.
4	Inflation Rate	Low	COE, NPC	COE falls 39% and NPC rises 22%; comparatively smaller impacts than other parameters.

#### Infrastructure and reliability sensitivity

**Fig. 29:**
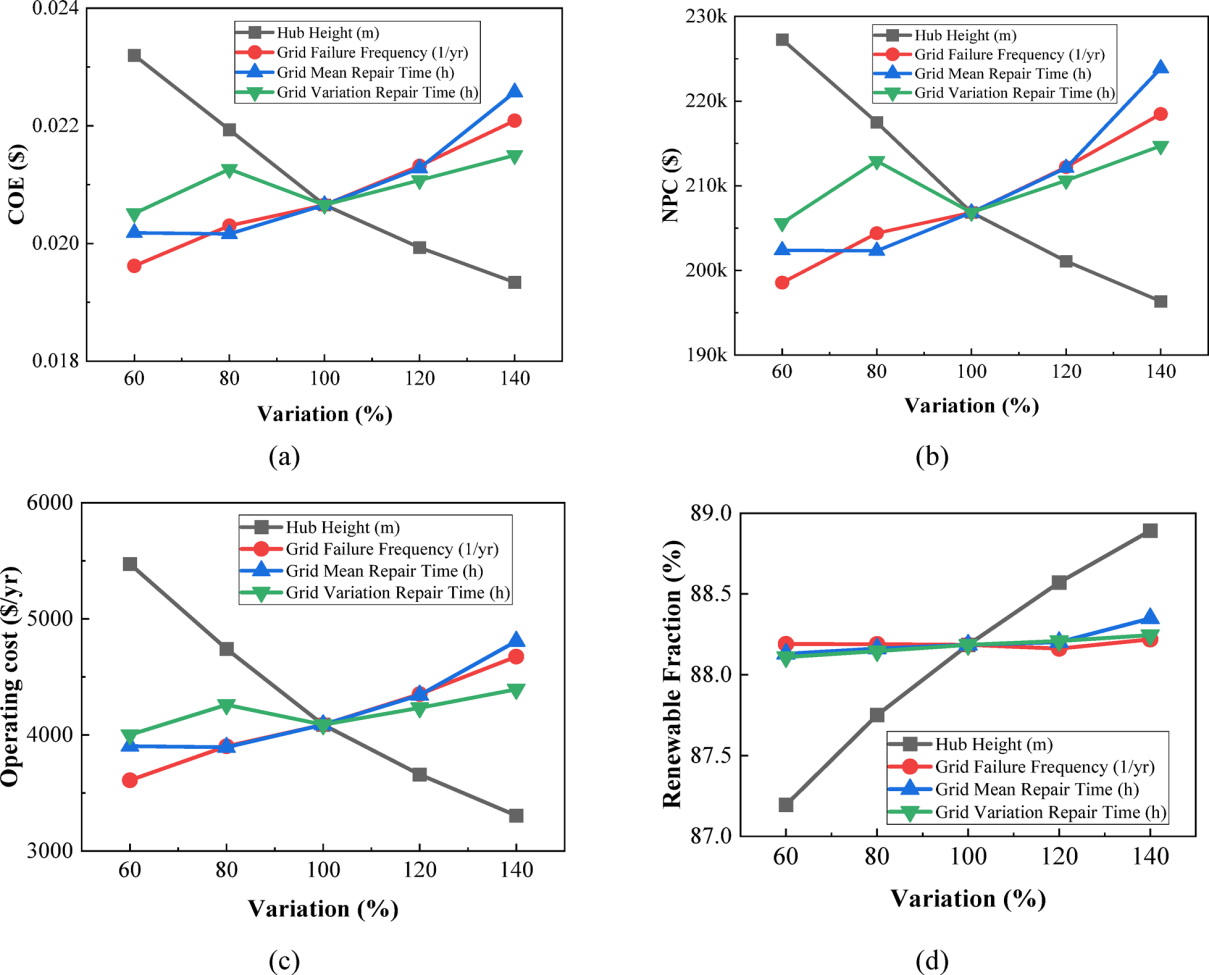
Impact of **(a)** COE, **(b)** NPC, **(c)** operating cost and **(d)** renewable fraction on infrastructure and reliability parameter variations.

The sensitivity analysis of infrastructure and reliability parameters in Fig. 29 demonstrates distinct and quantifiable impacts on system techno-economic performance across the 60–140% variation range. In Fig. 29(a), the COE decreases steadily with hub height, falling by 16.6% (0.02320→0.01934 $/kWh), whereas Grid Failure Frequency (GFF) increases COE by 12.6% (0.01962→0.02209 $/kWh). Mean Repair Time (MRT) produces a moderate 11.8% rise (0.02018→0.02257 $/kWh), while Repair Time Variation (RTV) increases COE by 4.8% (0.02051→0.02150 $/kWh). In Fig. 29(b), hub height again shows a declining trend, reducing NPC by 13.6% (227,280→196,347 $), while GFF increases NPC by 10.0% (198,553→218,478 $). MRT produces the strongest escalation, rising 10.6% (202,379→223,870 $), and RTV increases NPC by 4.4% (205,604→214,707 $). Operating cost patterns, in Fig. 29(c), mirror these behaviors: hub height causes a significant 39.6% reduction (5473→3305 $/yr), whereas GFF results in a 29.5% increase (3609→4674 $/yr), MRT grows by 23.1% (3903→4807 $/yr), and RTV rises by 9.8% (4001→4393 $/yr). Renewable fraction, in Fig. 29(d), remains comparatively stable, yet hub height enhances RF by 1.95% (87.19→88.89%), while MRT increases RF by 0.25% (88.13→88.35%). GFF and RTV exhibit minimal variations (< 0.1–0.2%). Overall, hub height shows the strongest positive techno-economic influence, substantially lowering costs while modestly increasing renewable penetration, whereas repair-related parameters (GFF, MRT, RTV) primarily raise COE, NPC, and operating cost, with only marginal effects on RF.

The sensitivity ranking Table 8 shows hub height as the dominant parameter, producing the largest reductions in COE, NPC, and operating cost. Grid Mean Repair Time and Failure Frequency follow, both significantly raising economic metrics due to increased downtime. Repair Time Variation exhibits the weakest impact, causing only minor fluctuations. Overall, infrastructure-related parameters influence economic performance more strongly than reliability variations, underscoring the technical importance of turbine height and grid stability.

**Table 8:** Sensitivity ranking of infrastructure and reliability parameters based on output variation.

Rank	Parameter	Sensitivity Level	Metrics Affected	Reason
1	Hub Height	Very High	COE, NPC, Operating Cost, RF	COE decreases by 16.6%, NPC decreases by 13.6%, and operating cost decreases by 39.6%. This indicates a clear influence on overall energy capture and system performance.
2	Grid Mean Repair Time	High	COE, NPC, Operating Cost, RF	COE increases by 11.8%, NPC increases by 10.6%, and operating cost rises by 23.1%, reflecting notable sensitivity to this parameter.
3	Grid Failure Frequency	Moderate	COE, NPC, Operating Cost	COE increases by 12.6%, NPC increases by 10%, and operating cost increases by 29.5%, showing a moderate but meaningful economic impact.
4	Repair Time Variation	Low	COE, NPC, Operating Cost, RF	COE increases by 4.8% and NPC increases by 4.4%, with only small variations in operating cost and renewable fraction, indicating limited overall effect.

#### Economic variation effects

All sensitivity analyses were performed using HOMER Pro’s re-optimization approach, allowing the system architecture and dispatch to adapt to parameter changes. Table 9 presents the sensitivity of system economics to ± 20% variations in the capital, replacement, and O&M costs of major components. Solar PV shows the highest influence, with NPC and COE shifting by ± 12.6%, indicating strong cost dependency. Wind turbines and BESS exhibit moderate sensitivity, with NPC and COE changes ranging between 2 and 3%. Converter costs also significantly impact performance, producing variations of − 4.8% to + 4.6% in NPC and − 5.3% to + 5.3% in COE. BioGen shows asymmetric behavior, where cost increases cause substantial reductions in NPC and COE (≈ − 10%), reflecting its nonlinear contribution to system economics. Negative NPC/COE variations indicate structural re-optimization, where higher BioGen cost reduces generator utilization and shifts energy supply toward PV, grid import, or storage.

**Table 9:** Impact of 20% variations in component costs on NPC and COE.

Components	Variations of Capital, Replacement and O&M cost	Variation of NPC (%)	Variation of COE (%)
Solar PV	20% (decrease)	−12.63%	−12.59%
	20% (increase)	+ 12.59%	+ 12.59%
Wind Turbine	20% (decrease)	−2.38%	−2.38%
	20% (increase)	+ 2.38%	+ 2.38%
BESS	20% (decrease)	−2.23%	−2.18%
	20% (increase)	+ 3.30%	+ 3.31%
BioGen	20% (decrease)	−0.45%	−2.72%
	20% (increase)	−9.96%	−9.79%
Converter	20% (decrease)	−4.82%	−5.33%
	20% (increase)	+ 4.60%	+ 5.26%

The sensitivity analysis Table 10 reveals that Solar PV exerts the strongest economic influence on the microgrid, with cost variations producing the highest changes in NPC and COE. The Converter ranks second due to its critical role in AC/DC power management, followed by the Wind Turbine, which shows moderate sensitivity. BESS exhibits low–moderate effects, reflecting its limited contribution to total system cost. BioGen demonstrates the lowest and irregular sensitivity, indicating minimal economic impact due to its smaller operational share.

**Table 10:** Sensitivity ranking of microgrid components based on the impact of cost variations on NPC and COE.

Rank	Component	Sensitivity Level	Reason
1	Solar PV	Very High	Produces the largest effect on system economics, with ± 12.6% change in NPC and COE for a ± 20% cost variation. As the primary generation source, its cost strongly drives overall economic performance.
2	Converter	High	A ± 20% cost shift causes − 4.82% to + 4.60% change in NPC and − 5.33% to + 5.26% in COE. Its key role in AC/DC conversion makes it economically influential despite smaller size relative to PV.
3	WT	Moderate	Cost variations lead to ± 2.38% changes in NPC and COE. WT contributes meaningfully to generation, giving it mid-level sensitivity.
4	BioGen	Low-Moderate	NPC changes − 2.23% to + 3.30%, COE − 2.18% to + 3.31%. Its cycling frequency and operational role create modest economic impact, but not as strong as generation sources.
5	BESS	Low	Exhibits small or irregular responses (NPC: −0.45% to − 9.96%, COE: −2.72% to − 9.79%), reflecting nonlinear effects. Although its cost increase sharply reduces NPC/COE, its overall influence remains small due to limited capacity share.

#### Solar and wind resource variability on optimal system configuration

Understanding how variations in solar irradiance and wind speed influence system performance is essential for validating the robustness of the selected hybrid configuration. To address this, a two-dimensional sensitivity analysis was conducted by varying the solar scaled average (2.8–6.0 kWh/m²/day) and wind scaled average (2.6–6.1 m/s) to examine changes in system optimality.

**Fig. 30:**
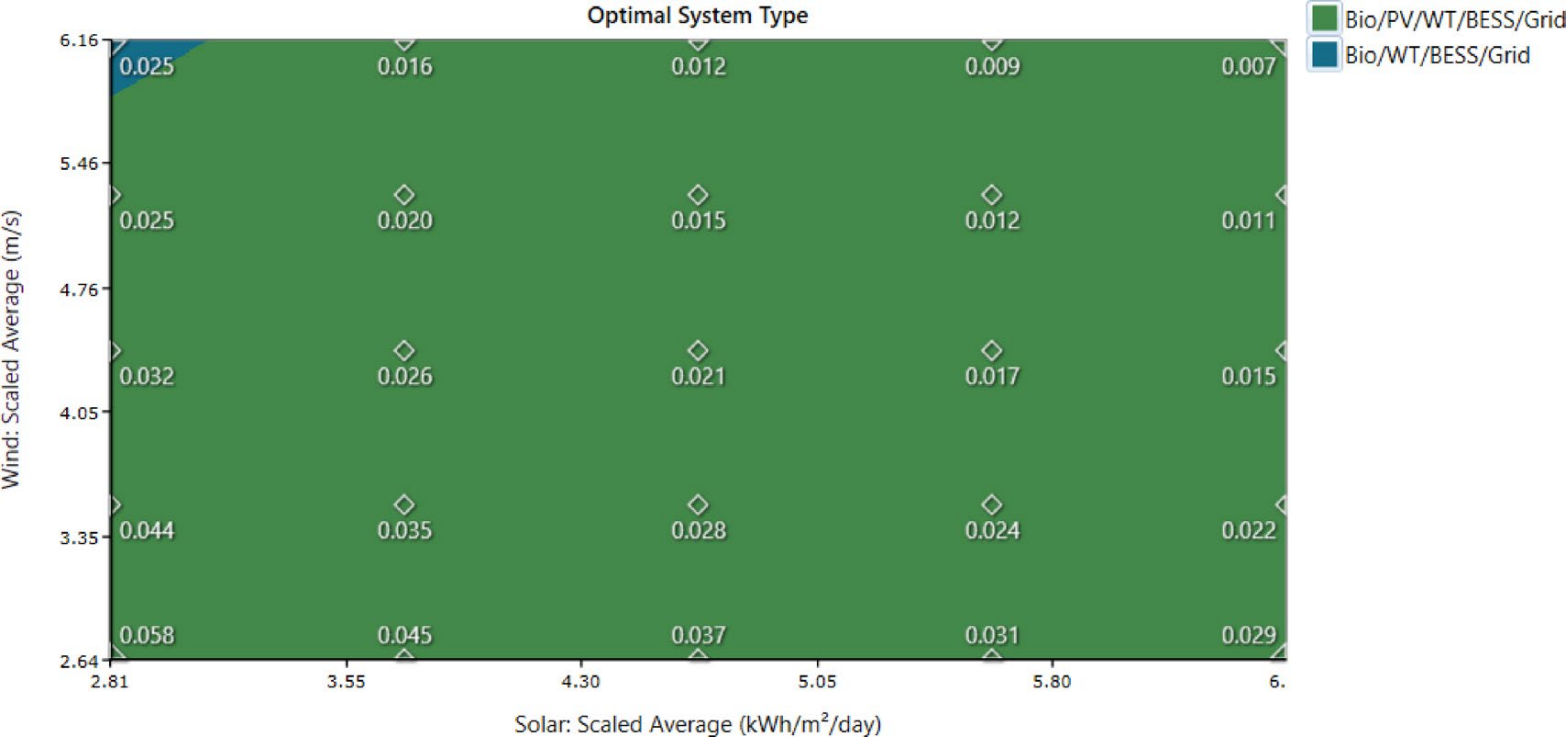
Optimal system sensitivity to solar–wind variations.

Figure 30 presents the sensitivity surface for the optimal system type under combined solar–wind resource fluctuations. Across nearly all sensitivity ranges, the hybrid Bio/PV/WT/BESS/Grid configuration remains dominant, indicating strong resilience of the selected optimal system. The cost of energy (COE) varies moderately across the sensitivity plane, ranging from 0.007 to 0.058 $/kWh, with the lowest COE observed at high solar (≈ 6.0 kWh/m²/day) and moderate wind speeds (≈ 4.7–5.0 m/s). Only a very small region at low solar (≈ 2.8 kWh/m²/day) and high wind (~ 6.1 m/s) shifts to the Bio/WT/BESS/Grid configuration, demonstrating that PV becomes less economical under extremely low solar conditions. Overall, the analysis confirms that the proposed system remains economically optimal under diverse climatic variations, reinforcing its suitability for long-term application in rural Bangladesh.

### Correlation analysis of microgrid variables

**Fig. 31:**
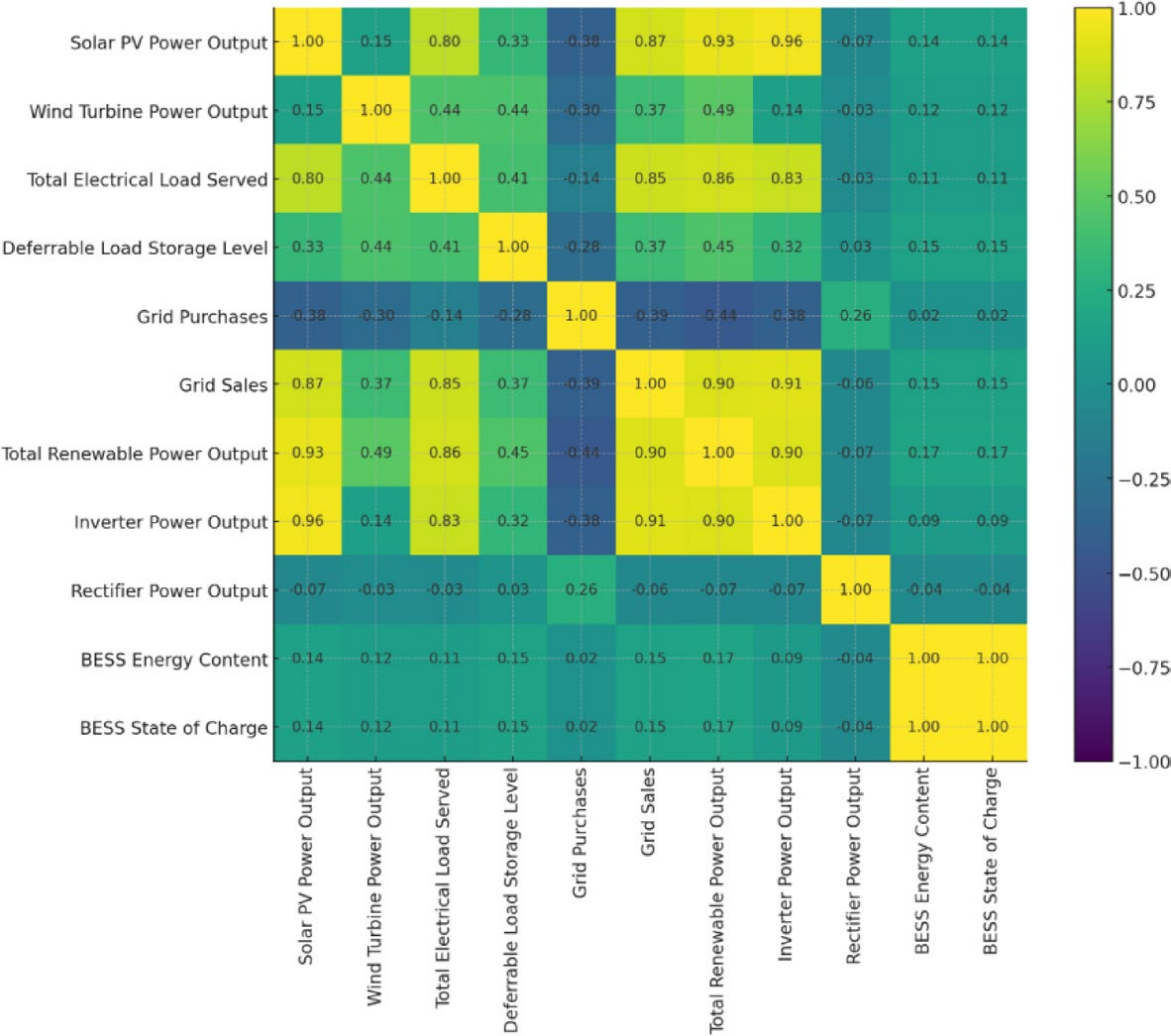
Correlation heatmap of microgrid power flow, storage, and grid interaction variables.

Figure 31 presents the correlation matrix capturing key operational interactions within the microgrid. Solar PV Power Output shows a very strong positive correlation with Inverter Power Output (*r* = 0.96), Total Renewable Power Output (*r* = 0.93), and Grid Sales (*r* = 0.87), reflecting that high PV generation directly increases renewable contribution and grid export. Wind Turbine Power Output exhibits moderate correlations with Total Renewable Power Output (*r* = 0.49) and Total Electrical Load Served (*r* = 0.44), indicating its secondary but supportive role. Total Electrical Load Served correlates strongly with Inverter Output (*r* = 0.83) and Renewable Output (*r* = 0.86), showing load-following behavior. Grid Purchases are negatively correlated with PV output (*r* = −0.38) and Grid Sales (*r* = −0.39), demonstrating the expected import–export trade-off. Battery State of Charge and Energy Content show very high mutual correlation (*r* = 1.00) but weak correlations with generation variables (≈ 0.10–0.15), indicating stable but less generation-driven storage dynamics.

### Practical deployment considerations

Practical implementation of the proposed hybrid microgrid requires consideration of biomass supply, policy, and maintenance feasibility. The biogas generator relies on locally available organic waste (~ 9 tonnes/day), including livestock manure and kitchen residues, which can meet demand year-round with organized collection. Policy barriers such as grid interconnection approvals and licensing for biogas plants exist; however, government incentives for decentralized renewable energy and rural electrification programs support deployment. Maintenance can be managed by trained local technicians, supported by modular system design and remote monitoring, ensuring reliable operation and long-term sustainability. The assumed biomass supply of 9 tonnes/day is based on local field data from households, livestock, and agricultural residues. Seasonal variations are minimal, as livestock and household organic waste are continuously generated. Competing uses for cooking or fertilizer are limited due to widespread access to alternative fuels and fertilizers. Organized collection and storage strategies ensure a sustainable feedstock supply for the microgrid throughout the year.

### BESS operation and utilization

The BESS in the optimal Case I configuration remains at high SOC (95–100%) for most of the time due to high PV and wind generation relative to load. Excess renewable energy is exported to the grid through net metering, reducing the need for battery discharge. The BESS is primarily designed for dispatchable backup during low generation periods and sudden load spikes, ensuring reliability rather than continuous daily cycling. This operational strategy balances cost-effectiveness and system security.

### Voltage and frequency response of the proposed microgrid

The dynamic voltage and frequency responses of the proposed grid-connected microgrid were evaluated to assess its stability under variations in renewable generation and load demand. The simulation was performed in MATLAB using a sensitivity-based linear dynamic model in which voltage and frequency deviations arise directly from instantaneous power imbalance. The dynamic voltage response is described by Eq. (24):

24 $$\:V\left(t\right)={V}_{\mathrm{nominal\:}}+{K}_{v}\cdot\:{\Delta\:}P\left(t\right)$$

and the dynamic frequency response can be expressed by Eq. (25) as:

25 $$\:f\left(t\right)={f}_{\mathrm{nominal\:}}+{K}_{f}\cdot\:{\Delta\:}P\left(t\right)$$

In this context, V(t) and f(t) denote the instantaneous PCC voltage (V) and system frequency (Hz). The nominal values are $\:{V}_{\mathrm{nominal\:}}=230\:V$and $\:{f}_{\mathrm{nominal\:}}$=50 Hz. The coefficients $\:{K}_{v}$ and $\:{K}_{f}$ represent the grid-influencing sensitivity factors; in this study, $\:{K}_{v}=0.05\mathrm{\:V/kW}$ and $\:{K}_{f}=0.0005\mathrm{\:Hz/kW}$ were selected to emulate the behavior of a moderately stiff grid. These values ensure that even a 100-kW imbalance produces only ≈5 V and ≈0.05 Hz deviation, keeping variations well within acceptable thresholds. The instantaneous power mismatch is shown in Eq. (26):

26 $$\:{\Delta\:}P\left(t\right)={P}_{\mathrm{renewable\:}}\left(t\right)-{P}_{\mathrm{load\:}}\left(t\right)$$

where $\:{P}_{\mathrm{renewable}}\left(t\right)$and $\:{P}_{\mathrm{load}}\left(t\right)$are the renewable generation and load demand at time $\:t$, respectively.

**Fig. 32:**
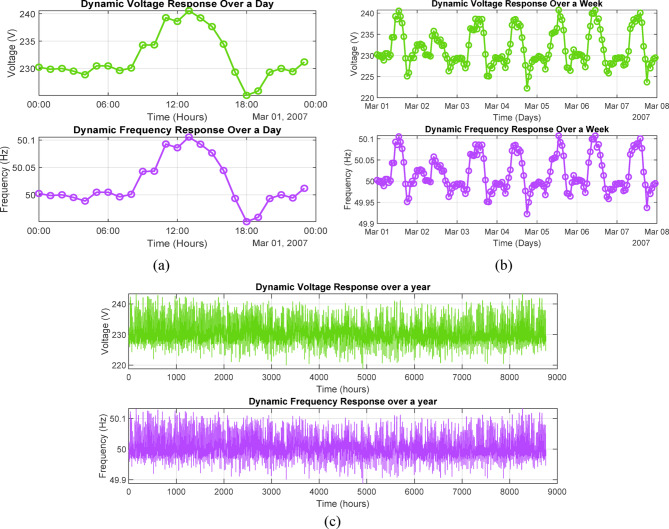
Variations in voltage and frequency of the proposed microgrid due to renewable energy integration: **(a)** daily fluctuations, **(b)** weekly fluctuations, and **(c)** annual fluctuations.

The resulting dynamic responses were evaluated across daily, weekly, and yearly horizons. Figures show smooth diurnal variations where voltage fluctuates between approximately 228–240 V and frequency varies within 49.95–50.15 Hz throughout the day. Weekly responses exhibit periodic behavior following recurring renewable patterns, with voltage remaining within 225–240 V and frequency between 49.90 and 50.12 Hz. The year-long response demonstrates dense variations caused by seasonal and hourly fluctuations, yet the voltage consistently stays within ± 10% of nominal (207–253 V), and frequency remains significantly tighter than the ± 0.5 Hz band allowed by standards. Figure 32 shows the simulated voltage and frequency deviations. Figure 32(a) presents the 24-hour response, Fig. 32(b) shows the one-week response, and Fig. 32(c) illustrates the one-year response, demonstrating stable microgrid operation under varying renewable penetration conditions.

In compliance with international standards such as IEEE 1547^141^ and IEC 62,116^142^, grid-connected systems must maintain voltage within ± 10% of nominal and frequency within 49.5–50.5 Hz during normal operation. Likewise, the Bangladesh Grid Code (BGC) requires frequency to remain within ± 0.5 Hz during steady-state and within ± 1 Hz during transient events, with voltage staying within ± 10% and recovering to this range within 1.5 s^143^. The simulated voltage and frequency deviations in this study remain well inside these regulatory limits across all time scales. This confirms that the proposed microgrid operates safely as a grid-supportive system, ensuring compatibility with national and international grid-code requirements while accurately capturing the dynamic influence of renewable energy variability.

### Comparison with others published work

Table 11 encapsulates the comparative global analysis of various renewable energy systems, structural configurations, geographic distributions, and categorical applications. It highlights the varying levels of renewable integration and economic metrics, reflecting context-specific adaptations to energy needs across rural, urban, and institutional settings. Comparing off-grid and on-grid systems, it places in relief the interplay of challenges and solutions specific to location in influencing the adoption of renewable energy. The variation in RF, NPC, and COE across diverse contexts represents a dynamic balance between technological feasibility, economic viability, and environmental sustainability. In the final analysis, the table shows a multi-dimensional view of renewable energy potential, tailored to the geographically and functionally distinct scenarios.

**Table 11:** Comparison of the proposed work with others published work.

System Structure	Location	System type and category	RF (%)	NPC ($)	COE ($/kWh)
PV-BioGen-BESS^144^	Punjab, India	Off-grid Village	100	$76,837	0.032
PV-BioGen-DG-WT-BESS^145^	Barwani, India	Off-grid City	100	170657.59	0.2899
PV-BioGen-DG-Grid-BESS^146^	Jhawani, Tezpur	On-grid Village	91	---	0.145
PV-BioGen-Grid-BESS^147^	Hattar, Pakistan	On-grid Industrial	25.2	135B PKR	14.1 PKR
PV-WT-BioGen-Grid BESS^148^	Shamshabad, India	On-Grid Residential	82	---	0.059
PV-BioGen-Grid^128^	Pabna, bangladesh	On-Grid Residential	80.1	321,798	0.0232
PV-WT-BioGen-Grid^149^	Rajshahi, Bangladesh	On-Grid Residential	59.4	46,813	0.0306
The Proposed Work	Rajbari, Bangladesh	On-Grid Residential and Commercial	88.19	206840.7	0.0207

The purpose of this study was to develop a realistic and comprehensive evaluation of a hybrid microgrid suitable for rural Bangladesh by combining technical feasibility, economic optimization, and multi-timescale operational stability in a single framework. The novelty of this work lies in its integrated modelling approach, where detailed residential, institutional, and irrigation load profiles were incorporated alongside converter compatibility checks and grid-connected performance analysis. Unlike studies relying only on steady-state metrics, this work includes daily, weekly, and yearly voltage–frequency simulations to verify stable operation under real renewable fluctuations, ensuring the system remains within acceptable limits of the Bangladesh Grid Code.

The study makes several important contributions. First, it identifies a well-balanced hybrid configuration capable of achieving a high renewable contribution of about 88%, demonstrating that coordinated use of PV, wind, BioGen, BESS, and limited grid support can significantly improve sustainability and reliability. Second, the economic evaluation shows that the optimized system maintains a low cost of energy (0.0207 $/kWh), highlighting its suitability for rural electrification. Third, the sensitivity analysis reveals that only a few components, particularly solar PV and converter units, exert notable influence on total system cost, giving planners clear guidance on which technologies require priority consideration in future deployments. Lastly, the dynamic voltage–frequency analysis confirms that the proposed microgrid consistently operates within the ± 10% voltage and ± 0.5 Hz frequency bands, validating its compliance with national grid-code requirements. Together, these findings provide a practical and robust pathway for designing resilient, cost-effective microgrids in the Bangladeshi context.

## Conclusions

This study presented a comprehensive techno-economic and dynamic stability assessment of a hybrid microgrid tailored for rural Bangladesh. Among eight evaluated configurations, the PV–WT–BioGen–BESS–Grid–Converter system (Case I) emerged as the optimal solution, achieving a high renewable fraction of 88.2% with the lowest net present cost (USD 206,841) and cost of energy (USD 0.0207/kWh). The results confirm that coordinated integration of solar, wind, biomass, and energy storage can reliably supply diversified rural loads while significantly reducing grid dependence and emissions. Sensitivity analysis identified solar PV cost as the dominant economic driver, while converter cost exhibited limited influence, demonstrating the robustness of the optimal configuration. Dynamic voltage–frequency simulations verified stable operation under varying resource and load conditions, with all deviations remaining within Bangladesh Grid Code and IEEE 1547 limits.

Future research will extend the present deterministic framework by incorporating stochastic resource modeling to explicitly capture solar irradiance and wind-speed uncertainty. Probabilistic optimization techniques—such as scenario-based Monte Carlo analysis and chance-constrained dispatch strategies—will be explored to improve system robustness under resource variability. Advanced energy management strategies will be developed using predictive control and demand-response mechanisms, particularly leveraging the flexibility of irrigation loads to enhance renewable utilization and reduce grid dependence. In addition, detailed electromagnetic transient (EMT) modeling using platforms such as PSCAD or MATLAB/Simulink will be conducted to validate inverter-level dynamics, protection coordination, and fault response. Finally, a field-level pilot implementation is recommended to validate long-term economic assumptions, component degradation behavior, and dynamic performance under real operating conditions, enabling refinement of the proposed framework for large-scale rural deployment.

## Data Availability

Data will be made available upon reasonable request from the corresponding author.

## Abbreviations

P: Electrical power (kW, W)
P_PV_: PV array output power(W)
P_PV,STC_: Rated PV power at STC (W)
G: Solar irradiance on PV surface (W/m²)
G_STC_: Solar irradiance at STC (1000 W/m²)
T_c_: PV cell temperature(°C)
T_c,STC_: Cell temperature at STC (25 °C)
T_a_: Ambient temperature(°C)
NOCT: Nominal operating cell temperature (°C)
$$\:{\alpha\:}_{p}$$: PV power temperature coefficient(%/°C)
$$\:{f}_{\mathrm{der\:}}$$: PV derating factor
$$\:\eta\:PV$$: PV efficiency (%)
$$\:{P}_{WT}$$: Wind turbine power output(kW)
V: Wind speed at hub height (m/s)
V_ref_: Wind speed at reference height (m/s)
H: Wind turbine hub height (m)
H_ref_: Reference height (m)
$$\:\alpha\:$$: Wind shear exponent
$$\:\rho\:$$: Air density (kg/m³)
$$\:{\rho\:}_{0}$$: Standard air density(kg/m³)
$$\:{C}_{p}$$: Wind turbine power coefficient
$$\:{P}_{\mathrm{Bio\:}}$$: Biogas generator output power (kW)
N_i_: Number of livestock of type i
f: Inflation rate (%)
N: Project lifetime (years)
m_i_: Manure produced per animal (kg/day)
M: Annual manure production(ton/year)
K_DM_: Dry matter fraction (%)
K_OM_: Organic matter fraction (%)
B_i_: Biogas yield (m³/ton)
V_b_: Annual biogas volume (m³/year)
K_e_: Biogas electrical efficiency
T_c_: Annual operating hours (h/year)
SOC: Battery state of charge (%)
SOC_min_: Minimum battery SOC (%)
SOC_max_: Maximum battery SOC (%)
$$\:\eta\:b$$: Battery efficiency (%)
E_b_: Battery energy capacity (kWh)
L_b_(t): Battery load at time t (kW)
$$\:\eta\:conv$$: Converter efficiency (%)
P_Grid_: Power exchanged with grid (kW)
E_grid,p_: Energy purchased from grid (kWh/year)
E_grid,s_: Energy sold to grid (kWh/year)
P_Load_: Electrical load demand (kW)
E_ren_: Renewable energy generation (kWh/year)
E_tot_: Total energy served (kWh/year)
C_ann_: Annualized system cost ($/year)
CRF: Capital Recovery Factor
i: Real discount rate (%)
i_n_: Nominal interest rate (%)
